# Ras GTPases Modulate Morphogenesis, Sporulation and Cellulase Gene Expression in the Cellulolytic Fungus *Trichoderma reesei*

**DOI:** 10.1371/journal.pone.0048786

**Published:** 2012-11-09

**Authors:** Jiwei Zhang, Yanmei Zhang, Yaohua Zhong, Yinbo Qu, Tianhong Wang

**Affiliations:** State Key Laboratory of Microbial Technology, School of Life Science, Shandong University, Jinan, China; University of Medicine & Dentistry of New Jersey - New Jersey Medical School, United States of America

## Abstract

**Background:**

The model cellulolytic fungus *Trichoderma reesei* (teleomorph *Hypocrea jecorina*) is capable of responding to environmental cues to compete for nutrients in its natural saprophytic habitat despite its genome encodes fewer degradative enzymes. Efficient signalling pathways in perception and interpretation of environmental signals are indispensable in this process. Ras GTPases represent a kind of critical signal proteins involved in signal transduction and regulation of gene expression. In *T. reesei* the genome contains two Ras subfamily small GTPases TrRas1 and TrRas2 homologous to Ras1 and Ras2 from *S. cerevisiae*, but their functions remain unknown.

**Methodology/Principal Findings:**

Here, we have investigated the roles of GTPases TrRas1 and TrRas2 during fungal morphogenesis and cellulase gene expression. We show that both TrRas1 and TrRas2 play important roles in some cellular processes such as polarized apical growth, hyphal branch formation, sporulation and cAMP level adjustment, while TrRas1 is more dominant in these processes. Strikingly, we find that TrRas2 is involved in modulation of cellulase gene expression. Deletion of *TrRas2* results in considerably decreased transcription of cellulolytic genes upon growth on cellulose. Although the strain carrying a constitutively activated *TrRas2^G16V^* allele exhibits increased cellulase gene transcription, the *cbh1* and *cbh2* expression in this mutant still strictly depends on cellulose, indicating TrRas2 does not directly mediate the transmission of the cellulose signal. In addition, our data suggest that the effect of TrRas2 on cellulase gene is exerted through regulation of transcript abundance of cellulase transcription factors such as Xyr1, but the influence is independent of cAMP signalling pathway.

**Conclusions/Significance:**

Together, these findings elucidate the functions for Ras signalling of *T. reesei* in cellular morphogenesis, especially in cellulase gene expression, which contribute to deciphering the powerful competitive ability of plant cell wall degrading fungi in nature.

## Introduction

Cellulose is one of the primary structural components in lignocellulosic materials that represent the major components of plant cell wall. Degradation of cellulose plays a key role in the global carbon cycle and this process mainly depends on the synergistic effects of many kinds of cellulolytic enzymes (e.g., cellobiohydrolases, endo-β-1, 4-glucanases and β-glucosidases) [1], [2]. Many filamentous fungi, such as *Trichoderma reesei*, could secret a broad range of cellulolytic enzymes which are needed in breakdown of cellulose to smaller, soluble sugars [3], [4], [5], [6]. With respect to the energy crisis and the global warming problems, conversion of cellulose to biofuels as an alternative fuel source has become the focus of world attention. To obtain a highly efficient cellulase complex used for biofuels production, the expression and secretion mechanism of cellulolytic enzymes has been subjected to study for decades [7], [8], [9], [10], [11]. Cellulase gene expression strictly depends on the induction by cellulose or its derivatives [12]. It is recognized that several transcriptional factors, e.g., the transcriptional activators XlnR/Xyr1 [13], Ace2 [14], and the HAP2/3/5 complex [15] as well as the repressors CreA/Cre1 [16], Ace1 [17], are involved in the transcriptional regulation of cellulase genes. However, little is known about the signal pathway that cells sense and transmit extracellular cellulose signal to stimulate the transcription of cellulolytic enzyme genes.

Wang and Nuss [18] discovered that the expression of cellobiohydrolase I gene was regulated by a GTP-binding-protein-linked signalling pathway in the fungal pathogen *Cryphonectria parasitica*. In *T. reesei*, stimulation of cellulolytic enzyme gene expression by light involves the function and activation of the G-Alpha Proteins GNA1 and GNA3 [19]. Cellulase induction in *T. reesei* by sophorose can be enhanced by increasing intracellular cAMP levels, which implies that cAMP signalling pathway probably modulates the transcription of cellulase genes [20]. The lower number of cellulase and hemicellulase encoding genes in the genome of *T. reesei* than other sequenced biomass-degrading fungi also reveals that adequate signal transduction machineries are required during regulation of sensing cellulose [21].

Ras-like proteins are members of Ras subfamily of GTPase proteins that function as the molecular switch through cycling between inactive GDP-bound and active GTP-bound forms, which play important roles in various signal transduction pathways controlling cell proliferation, morphogenesis, oncogenic transformation, vesicular trafficking and gene expression [22], [23], [24]. In *Saccharomyces cerevisiae*, Ras1 and Ras2 sense extracellular glucose to regulate cell cycle progress through cAMP signalling pathway, and Ras2 also controls pseudohyphal differentiation via both MAPK and cAMP pathways [25], [26]. Expression of the dominant activated *Ras2* allele triggers filamentous growth in maize pathogen *Ustilago maydis*, while similar change in cell morphology was not found in the Ras1 dominant activated strain. Moreover, both Ras1 and Ras2 are involved in inducing pheromone gene expression in this fungus [27], [28]. In human pathogens *Candida albicans* and *Cryptococcus neoformans*, Ras1 has been shown to control filamentation and virulence via MAP kinase and cAMP-PKA signalling pathway [29], [30]. Through a TBLASTX search using nucleotide sequences of *S. cerevisiae* Ras1 and Ras2 as queries, we found two putative homologues of the Ras-subtype GTPase, TrRas1 and TrRas2, in the genome of the cellulolytic model fungus *T. reesei*. However, detailed functions of these two Ras signal proteins in this organism remain unknown.

It is also known that in many fungi, e.g., *S. cerevisiae*, *C. neoformans* and *C. albicans*, Ras plays an important role in activating the cAMP pathway to regulate cell morphology and cell cycle [32]. Meanwhile, Schuster *et al*. [31] discovered that two crucial components of cAMP pathway, adenylate cyclase and protein kinase A, were involved in light modulated cellulase gene expression and regulation of vegetative growth in *T. reesei*. With respect to the knowledge mentioned above, it would be attractive to explore the role of Ras signalling in the regulation of morphological development and cellulase gene expression and to study the relationship between Ras and cAMP pathway in *T. reesei*. In this work, we have found that both TrRas1 and TrRas2 play similar roles in morphogenesis and adjusting cAMP level, while TrRas1 is more dominant than TrRas2. Moreover, we also provide the evidence that TrRas2 is involved in regulation of cellulase gene expression.

## Results

### Characterization of TrRas1 and TrRas2

Inspection of *T. reesei* genome sequences with TBLASTX revealed two putative Ras GTPases, named TrRas1 and TrRas2, with high homology to *S. cerevisiae* Ras1 and Ras2. The corresponding *TrRas1* gene (GenBank accession no. JX114947) consists of a predicted 967 bp open reading frame interrupted by three introns and encodes a protein of 215 amino acids, which shares 41.0% and 39.3% amino acid sequence identity to *S. cerevisiae* Ras1 and Ras2 respectively. While the putative 237-amino-acid TrRas2 protein (GenBank accession no. AFQ23948) is encoded by a 994 bp open reading frame interrupted by a 280 bp intron, which has the identity of 31.4% and 30.5% to *S. cerevisiae* Ras1 and Ras2 respectively. Sequencing of *TrRas1* and *TrRas2* cDNA from RT-PCR confirmed the model provided above. Transcripts of *TrRas1* and *TrRas2* could be detected in both conidiospores and hyphal cells (data not shown).

**Figure 1:**
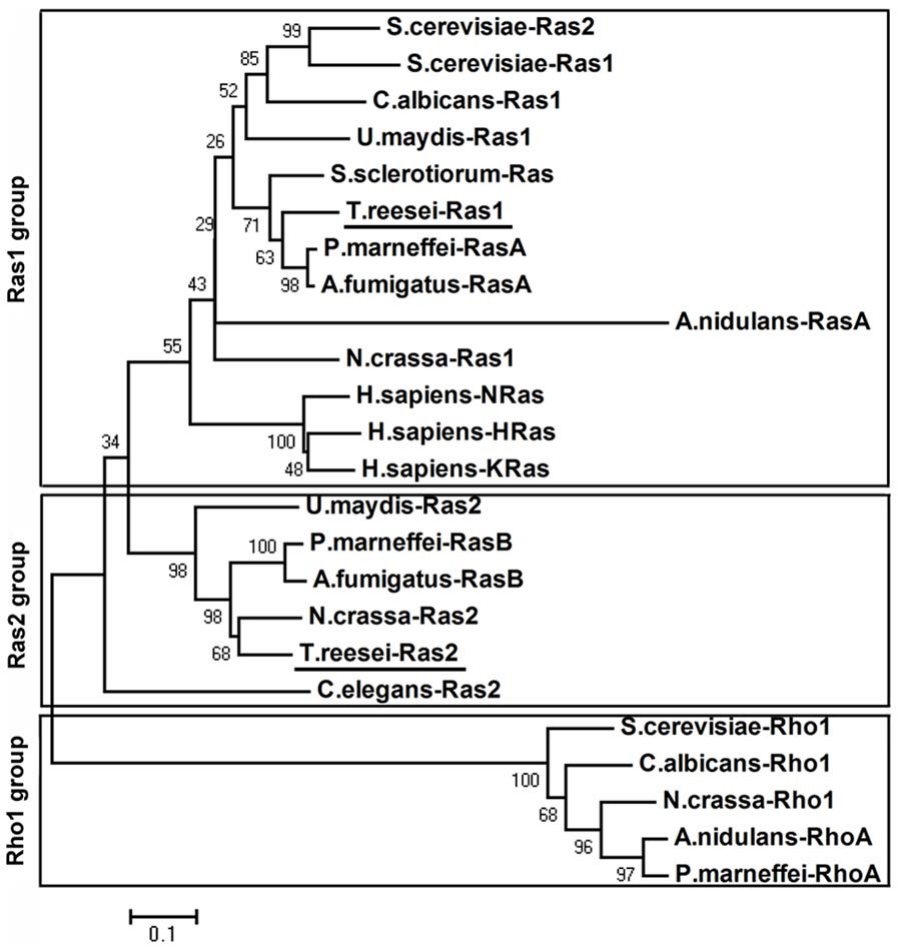
Phylogenetic analysis of Ras proteins. The Ras-related Rho orthologues were used as an outgroup. The analysis was performed using Neighbor-joining method in the MEGA4.0 software and 1000 Bootstrap replications as test of phylogeny. GenBank accession numbers for the proteins are as follows: S. cerevisiae-Ras1, AAA34958; S. cerevisiae-Ras2, AAA34959; C. albicans-Ras1, AAF03566; U. maydis-Ras1, AAO19640; U. maydis-Ras2, AAO19639; S. sclerotiorum-Ras, AAT75139; P. marneffei-RasA, AAO64439; P. marneffei-RasB, EEA25931; A. fumigatus-RasA, EAL91488; A. fumigatus-RasB, EAL93074; A. nidulans-RasA,; N. crassa-Ras1, CAA37612; N. crassa-Ras2, BAA03708; H. sapiens-HRas, AAM12630; H. sapiens-KRas, AAB41942; H. sapiens-NRas, AAA60255; C. elegans-Ras2, CAA84796; T. reesei-Ras1, EGR51722; T. reesei-Ras2, EGR45548; S. cerevisiae-Rho1, AAA34977; N. crassa-Rho1, ACD01425; C. albicans-Rho1, XP_715825; A. nidulans-RhoA, AAK08118; P. marneffei-RhoA, XP_002144340.

Alignment of the amino acid sequences of TrRas1 and TrRas2 along with those of their orthologues of other fungi revealed that all the conserved domains, GTP or GDP binding site, GAP effector binding site, the GTPase domain and the CAAX box for membrane association [22], are included in both TrRas1 and TrRas2. The amino acid sequence of TrRas1 has high identity to the Ras1/RasA proteins of Penicillium marneffei (81.9%), *Aspergillus fumigatus* (81.9%), *Sclerotinia sclerotiorum* (78.8%), *U. maydis* (71.0%) and *Neurospora crassa* (67.6%), while TrRas2 shares great identity to the Ras2/RasB proteins from *N. crassa* (72.3%), P. marneffei (67.1%), *A. fumigatus* (65%) and *U. maydis* (57.8%). As shown in Figure 1, phylogenetic analysis using Ras-related Rho orthologues as an outgroup demonstrated that TrRas1 and TrRas2 cluster well with the corresponding Ras proteins from other organisms. The designations of TrRas1 and TrRas2 were chosen based on their subclass loci in the phylogenetic tree.

### Transcription of *TrRas1* and *TrRas2* is not induced by specific carbon source

**Figure 2:**
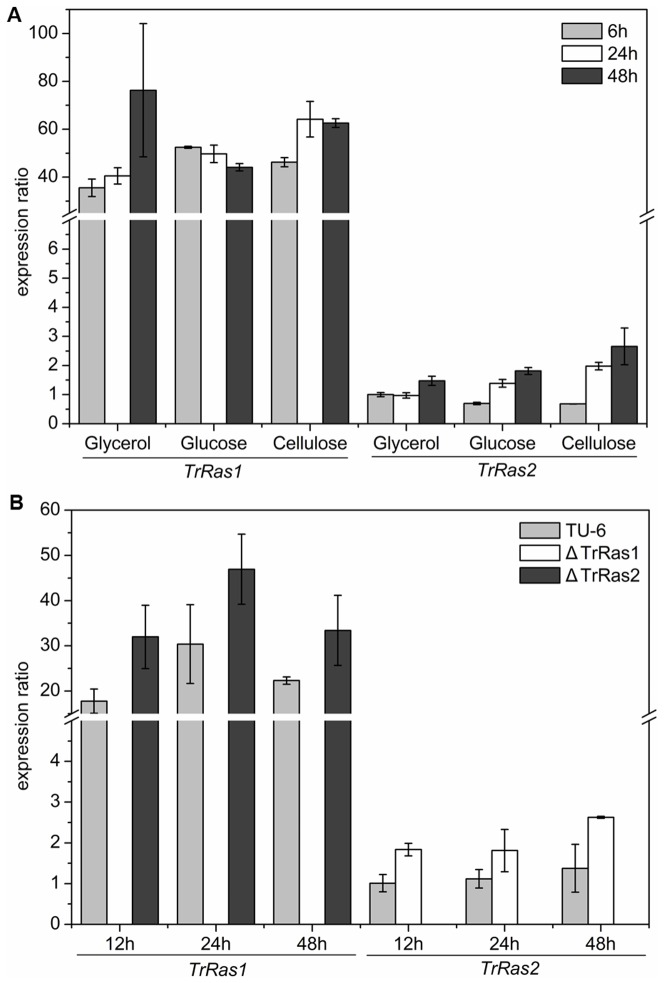
Analysis of transcript levels of *TrRas1* and *TrRas2* mRNA by qRT-PCR. (A) Transcript abundances of *TrRas1* and *TrRas2* on different carbon sources. The ratios of the expression of *TrRas1* and *TrRas2* to that of the actin reference gene were calculated in *T. reesei* QM9414 cultured on glycerol, glucose or cellulose. The relative mRNA levels were presented by setting the amount of *TrRas2* mRNA detected on glucose at 6 h as 1. (B) Quantitative real-time PCR analysis of *TrRas1* or *TrRas2* mRNA levels in the Δ*TrRas2* or Δ*TrRas1* mutant. The ratios of the expression of *TrRas1* and *TrRas2* to that of the actin reference gene were calculated. The relative mRNA levels were presented by setting the amount of *TrRas2* mRNA in *T. reesei* TU-6 detected at 12 h as 1. Values are means of three independent experiments. Error bars represent standard deviations.

In order to be able to relate the Ras signalling proteins to carbon source utilization, the transcription of *TrRas1* or *TrRas2* itself on various carbon sources was firstly investigated. To this end, the strain QM9414 was pre-grown with glycerol, and then the mycelia were transferred to liquid minimal medium with glycerol, glucose or cellulose as the sole carbon source and incubated for 6, 24 or 48 h. No significant differences in *TrRas1* transcript formation were observed for the cellulase-repressing substance glucose and for the cellulase-inducing carbon source cellulose. These transcript levels never exceed those observed on glycerol. Meantime, statistical analyses also revealed the similar expression levels of *TrRas2* for three different carbon sources (Figure 2A). These data strongly indicate that the transcriptions of *TrRas1* and *TrRas2* are neither significantly influenced by glucose repression nor by cellulose induction.

TrRas1 has high amino acid identity (45.5%) to TrRas2, which indicates that they may possess overlapping functions in the cellular processes. To support this, we have detected the transcriptional abundance of *TrRas1* or *TrRas2* gene in Δ*TrRas2* or Δ*TrRas1* mutant strain respectively. From the data, we found that transcript of *TrRas1* in the Δ*TrRas2* mutant increased by 50%–80% compared to the wild-type strain, while *TrRas2* in the Δ*TrRas1* strain increased by 60%–90% (Figure 2B). In addition, *TrRas1* and *TrRas2* showed significant differences in mRNA levels regardless of which carbon source was used in media, reaching more than 75-fold enhanced transcript abundance of *TrRas1* compared to *TrRas2* (Figure 2A). Similar phenomena was found in *Mucor racemosus*, in which the different transcript levels between *MRas1* and *MRas3* were observed, suggesting these two proteins play distinct roles during morphogenesis in this fungus [33]. These data suggest that TrRas1 and TrRas2 may play distinct roles in addition to overlapping functions in *T. reesei*.

As mentioned above, transcriptions of Ras GTPases TrRas1 and TrRas2 themselves do not respond to certain carbon source signals. Nevertheless, it cannot be rejected that they are still involved in signalling pathways by which *T. reesei* cells regulate carbon source sensing. Consequently, their putative functions in carbon source utilization and morphogenesis would be investigated in the next studies through gene targeting technology.

### Growth of mutants, Δ*TrRas1* and Δ*TrRas2*, on different carbon sources

Null mutation at the *TrRas1* or *TrRas2* locus was introduced into the parental strain TU-6 by gene replacement using *ptrA* or *pyrG* gene [34], [35] as the selective marker respectively. No putative other genes were contained in the flanking regions of the deletion cassette to ensure that only the relevant target genes were replaced. PCR and Southern blot analysis revealed that *TrRas1* or *TrRas2* was indeed deleted without ectopic integration of the deletion cassette (Figure S1B, C; Figure S2B, C). Detection of mRNA expression by RT-PCR further confirmed the absence of *TrRas1* or *TrRas2* transcript in relevant mutants (Figure S1D; Figure S2D).

**Figure 3:**
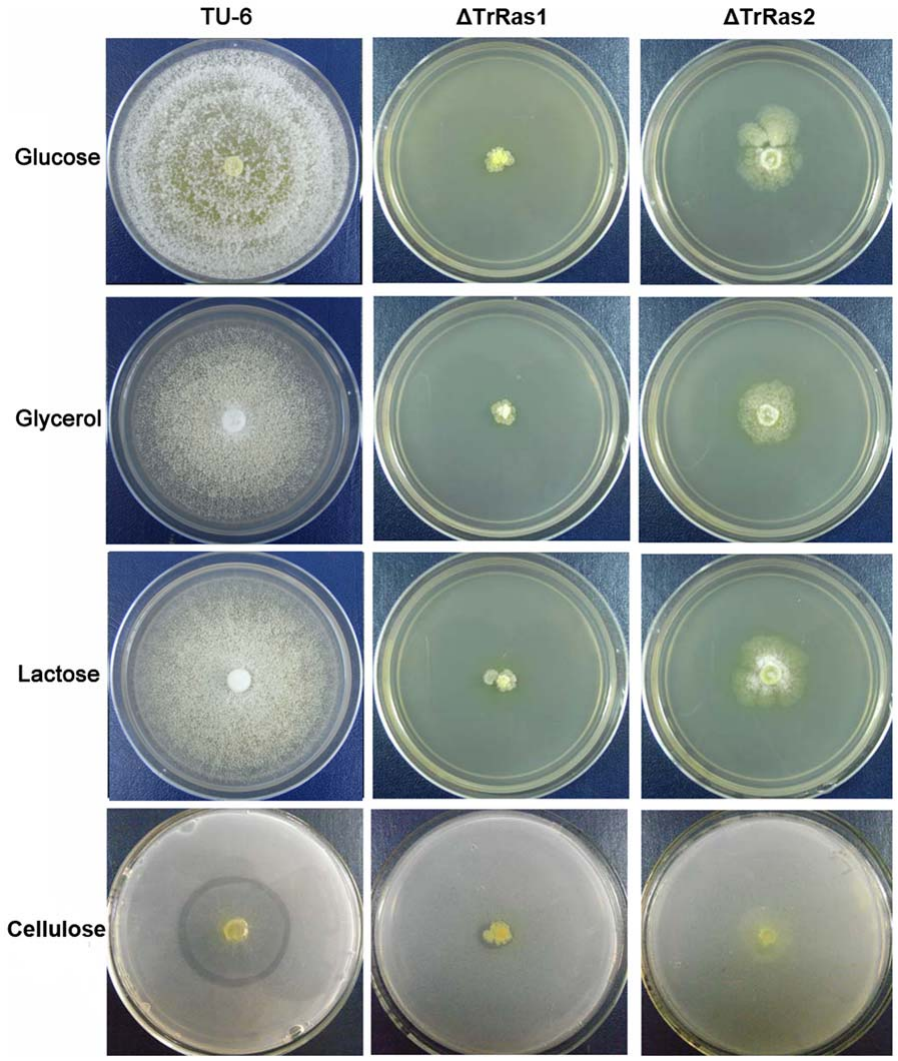
Growth of TU-6, Δ*TrRas1* and Δ*TrRas2* on different carbon sources. Strains were grown on plates containing minimal medium supplemented with 1% glucose, glycerol, lactose or cellulose at 30°C for 4 days.

To examine the influence of TrRas1 and TrRas2 on carbon source utilization and morphological development, a series of growth experiments were conducted in agar plates. In detail, the parental strain TU-6 and the deletion strains Δ*TrRas1* and Δ*TrRas2* were cultured on MM plates containing glucose, glycerol, lactose or cellulose for 4 days. The results were shown in Figure 3. Both mutants showed dramatically reduced colony size on all carbon sources investigated in this work while the parental strain was able to form the normal colonies, indicating that the growth of *T. reesei* on agar plate is directly influenced by deletion of *TrRas1* or *TrRas2* no matter what carbon source is used in the medium. Moreover, disruption of TrRas1 results in a more severe growth deficiency than that of TrRas2, suggesting that TrRas1 plays more dominant regulatory roles in fungal development. Interestingly, Δ*TrRas2* could not produce clear zone around the colony on cellulose plate as compared with the parental strain and Δ*TrRas1*, which suggests that TrRas2 may be involved in regulation of cellulase production.

### TrRas1 is essential for polarized apical growth, branching and sporulation

**Figure 4:**
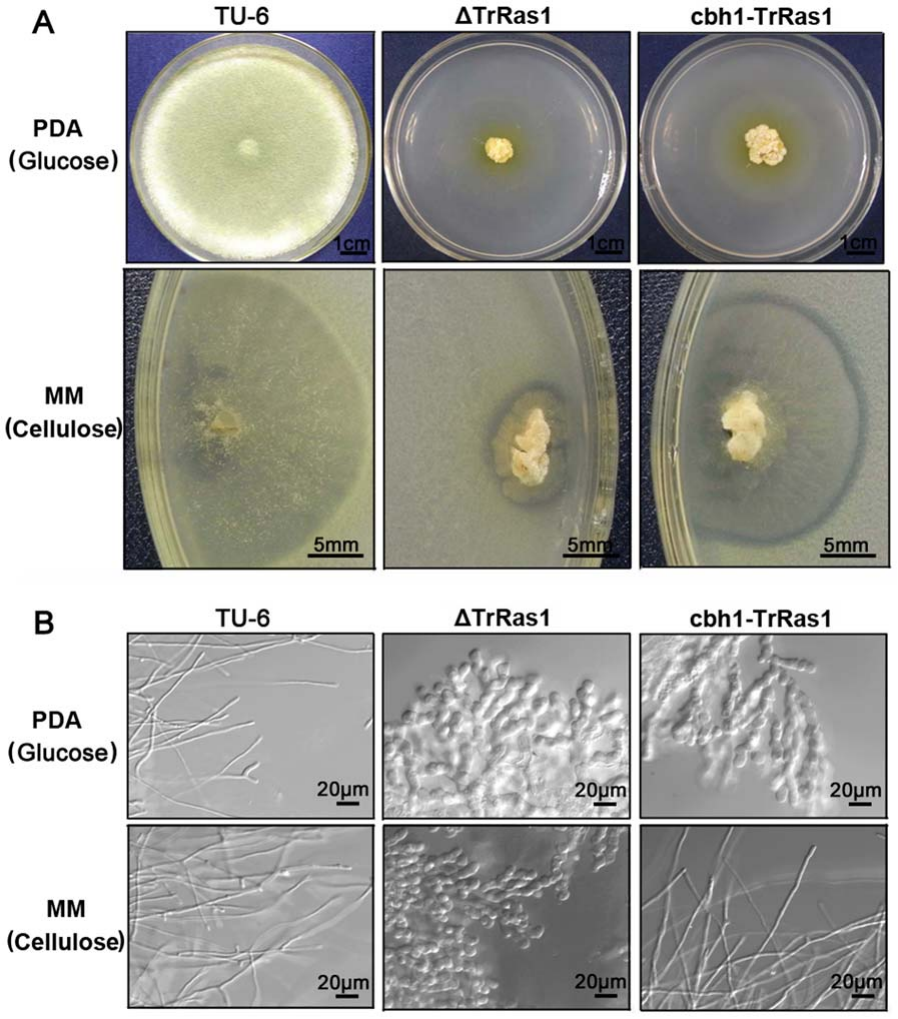
TrRas1 regulates filamentation and sporulation in *T. reesei*. The colonial phenotypes (A) and hyphal morphology (B) of strains TU-6, Δ*TrRas1* and *cbh1-TrRas1* cultured on PDA plates (glucose) or MM plates (cellulose) for 5 days. The microscopic images were captured using DIC. Scale bars are shown in the figure.

When cultured on complete medium PDA (supplemented with glucose as the carbon source), the Δ*TrRas1* mutant formed dramatically decreased colonies with dense mycelia and no conidiospores (Figure 4A) which was similar to that observed on minimal medium agar plate in Figure 3. Retransformation experiment cannot be carried out for the Δ*TrRas1* mutant due to the severe growth defects of disruption of *TrRas1*. Therefore, we constructed the *cbh1-TrRas1* mutant, in which the native *TrRas1* promoter was replaced with the cellulose-inducible *T. reesei cbh1* promoter, through homologous recombinant experiments to further confirm the role of TrRas1 in fungal development. The *cbh1-TrRas1* transformant was confirmed by PCR and Southern blot analysis (Figure S3B, C, D). The same as Δ*TrRas1*, *cbh1-TrRas1* showed no sporulation and small and dense colony that could not expand under repressing conditions (glucose), whereas it returned to the wild-type colonial phenotypes when shifted to inducing conditions (cellulose) (Figure 4A). Although null mutation of TrRas1 results in severe defects on morphogenesis and sporulation, the Δ*TrRas1* strain still maintains the ability in degrading cellulose to form clear zone (Figure 4A).

The *TrRas1* mutants were examined microscopically to assess whether there were defects in hyphal morphogenesis. In contrast to the parental strain TU-6, Δ*TrRas1* exhibited highly branched, swollen and misshapen hyphal cells after 5 days growth on plates containing either glucose or cellulose (Figure 4B). As expected, when cultured on glucose medium, the *cbh1-TrRas1* mutant displayed the same hyphal morphological defects as in Δ*TrRas1*, whereas it became to normal filamentous fungal cells under cellulose-inducing conditions (Figure 4B). Taken together, these results suggest that (a) TrRas1 is an important signal protein that controls hyphal cell formation and polarized apical growth in *T. reesei*; (b) deficiency of the hyphal growth subsequently results in a small and dense colony in the *TrRas1* deletion mutant; (c) TrRas1 is involved in controlling asexual development in *T. reesei*. Although similar roles of Ras1 during filamentous growth have been reported in many ascomycetous fungi, e.g., *U. maydis* and *P. marneffei* [28], [37], deletant of this GTPase could not be generated in these fungi because such a mutation might be either lethal or leads to severe defects. In this work, deletion of *TrRas1* was successfully carried out and resulted in severe defects in either hyphal polarized apical growth or sporulation in *T. reesei*, which provide the new evidence that Ras1 is essential for hyphal growth in ascomycetous fungi.

### TrRas2 also modulates polarized apical growth and hyphal branch formation

**Figure 5:**
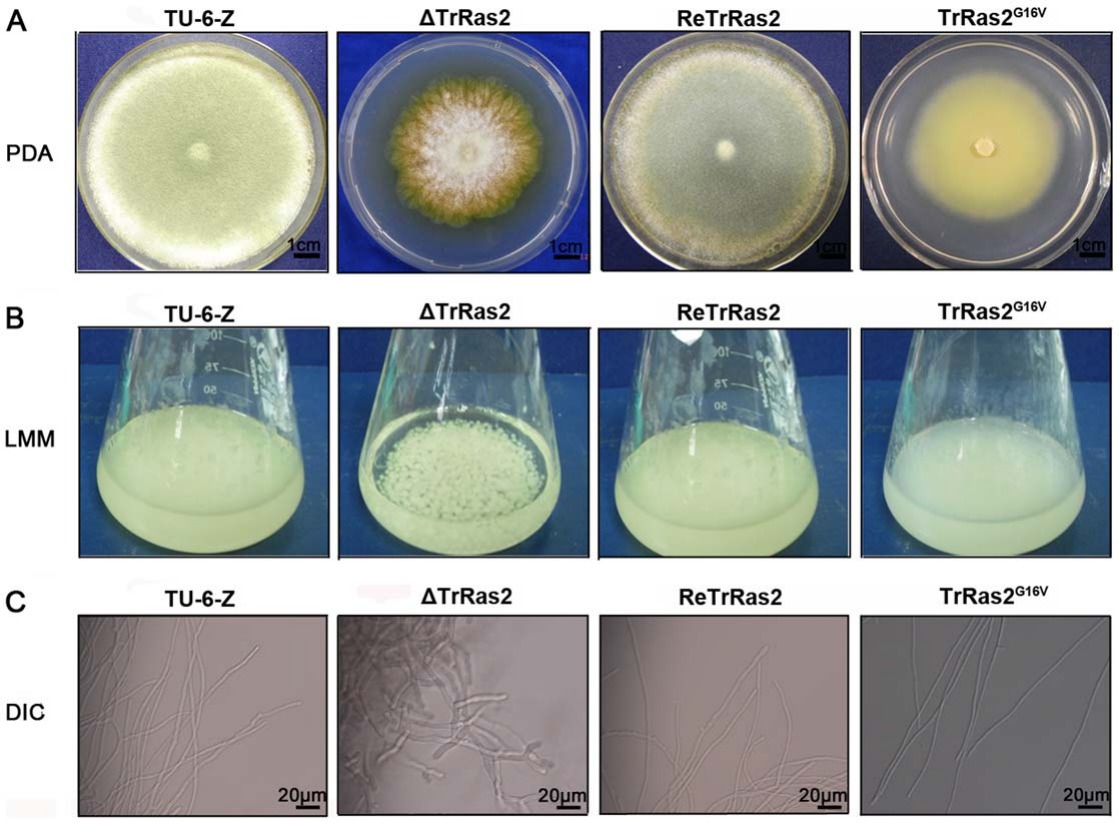
TrRas2 modulates polarized apical growth, branch formation and sporulation in *T. reesei*. Morphological phenotypes of strains TU-6-Z, Δ*TrRas2*, Re*TrRas2* and *PAnigpdA*-*TrRas2^G16V^* on PDA plates (A) and in liquid minimal medium (LMM) (B). Strains were cultured on PDA plates for 5 days at 30°C or were grown in LMM supplemented with 2% glucose as the carbon source for 48 h. (C) Hyphal phenotypes of *TrRas2* strains in LMM. The microscopic images were captured using DIC. Scale bars are shown in the figure.

*T. reesei* strain with null mutation in the *TrRas2* gene displays reduced growth rate in comparison to the parental strain TU-6 on minimal medium agar plates. Meanwhile, we have also found that the Δ*TrRas2* strain showed decreased cellulase activity on cellulose plate (Figure 3). To genetically confirm the phenotypes in the Δ*TrRas2* mutant, retransformation of *TrRas2* was carried out as described in the methods. PCR and RT-PCR analysis revealed the regain of *TrRas2* (Figure S2D, E). Phenotype detection revealed that Re*TrRas2* could completely complement the defects of the Δ*TrRas2* mutant and grow similarly to wild-type (Figure 5A, B, C). In addition, to study the functions of TrRas2 in regulation of morphogenesis and cellulose-cellulase signalling pathway in detail, we constructed a mutant strain *PAnigpdA*-*TrRas2^G16V^* which carried an active *TrRas2* allele (*TrRas2^G16V^*) whose product was defective in GTPase activity, thus resulting in permanent signal transmission. In many fungi, such mutations have been considered dominant over the wild-type allele in vivo and applied to study the function of Ras GTPase [27], [29], [36], [37]. PCR and Southern analysis revealed the insertion of *gpdA(p)-TrRas2^G16V^* as single-copy integration in the genome of TU-6 (Figure S4B, C, D). Sequencing of the *TrRas2* cDNA from the *PAnigpdA*-*TrRas2^G16V^* strain indicated the successful expression of the mutant allele (data not shown). Transcription of the *TrRas2* gene in the *PAnigpdA*-*TrRas2^G16V^* mutant was analyzed under cellulose-inducing conditions. Statistical analysis by quantitative real-time PCR showed 7.7-, 33.8- and 13.8-fold up-regulation of the *TrRas2* gene transcript in the *PAnigpdA*-*TrRas2^G16V^* mutant compared to the parental strain after inducing for 9 h, 20 h and 40 h respectively (Figure S4E). This increased transcript accumulation in the mutant may be because the expression of *TrRas2^G16V^* allele is under the control of strong *AnigpdA* promoter. Boyce *et al*. have found that overexpression of *RasA* did not affect the phenotypic effects of the mutant *RasA^G19V^* allele in *P. marneffei* [37]. Indeed, the dominant activated *TrRas2^G16V^* mutant strain which expresses *TrRas2^G16V^* under control of its endogenous promoter displays similar morphological phenotypes as in *PAnigpdA*-*TrRas2^G16V^* (as shown in Text S1 and Figure S6). Therefore, it is believed that the phenotypic changes in the *PAnigpdA*-*TrRas2^G16V^* transformant were attributed to the activated form of *TrRas2^G16V^* but not the overexpression of this gene.

The effects of TrRas2 on morphological phenotypes were analyzed by culturing the parental strain TU-6-Z and relevant mutants on PDA plates or liquid minimal medium (LMM) with glucose as the carbon source. The parental strain TU-6-Z, which was constructed by transforming TU-6 with the *pyrG* cassette and could be cultured independent of uracil, was applied as a control in functional analysis of TrRas2. Neither the growth and morphological phenotype nor the cellulase activity is affected by the integration of the *pyrG* cassette in the TU-6-Z transformant (data not shown). After 5 days growth on PDA plates, colonies of the parental strain TU-6-Z comprising hyphae and conidia were visible. In contrast, the Δ*TrRas2* strain exhibited reduced colonies with irregular boundaries and greatly decreased aerial hyphae (Figure 5A). Despite no spores were found in the Δ*TrRas2* strain on PDA plate at early stages, conidiation was visible after 9–10 days growth (data not shown). Interestingly, the dominant activated *PAnigpdA*-*TrRas2^G16V^* strain also showed reduced colonies with no conidia and aerial hyphae, but their colonies exhibited regular borders (Figure 5A). In contrast to the wild-type, the Δ*TrRas2* mutant formed aggregated hyphae with a hyper-branching phenotype while dominant activation of the TrRas2 resulted in more dispersive hyphae with fewer branches and enhanced polarized apical growth when cultured in LMM (Figure 5B, C). These findings are similar to those in *N. crassa* and *A. fumigatus*, in which disruption of Ras2/RasB results in a series of phenotypes, e.g., decreased conidiation, reduced hyphal growth rate on agar plate, irregular colonial boundaries and increased branching [46], [47]. Obtained data on the phenotypes of the TrRas2 mutants suggest that correct cycling between GDP-bound and GTP-bound TrRas2 is required for growth. Although growth rates are both delayed for Δ*TrRas2* and *PAnigpdA*-*TrRas2^G16V^* strains on either PDA plates or LMM, the biomass of Δ*TrRas2* could reach the similar level as that of the parental strain after 60 h of growth in LMM while that is not for *PAnigpdA*-*TrRas2^G16V^* (Figure S5A, B). Similarly, deletion of RasB does not influence the total hyphal mass accumulation in liquid culture in *A. fumigatus* [46]. The fewer biomass accumulations in the *PAnigpdA*-*TrRas2^G16V^* mutant may be due to the decreased rate of branch formation. These results demonstrate that TrRas2 also acts to modulate polarized apical growth and branch formation in *T. reesei*, albeit the morphological defects are clearly less severe than that of Δ*TrRas1* mutant.

### Cellulase formation is influenced by TrRas2

**Figure 6:**
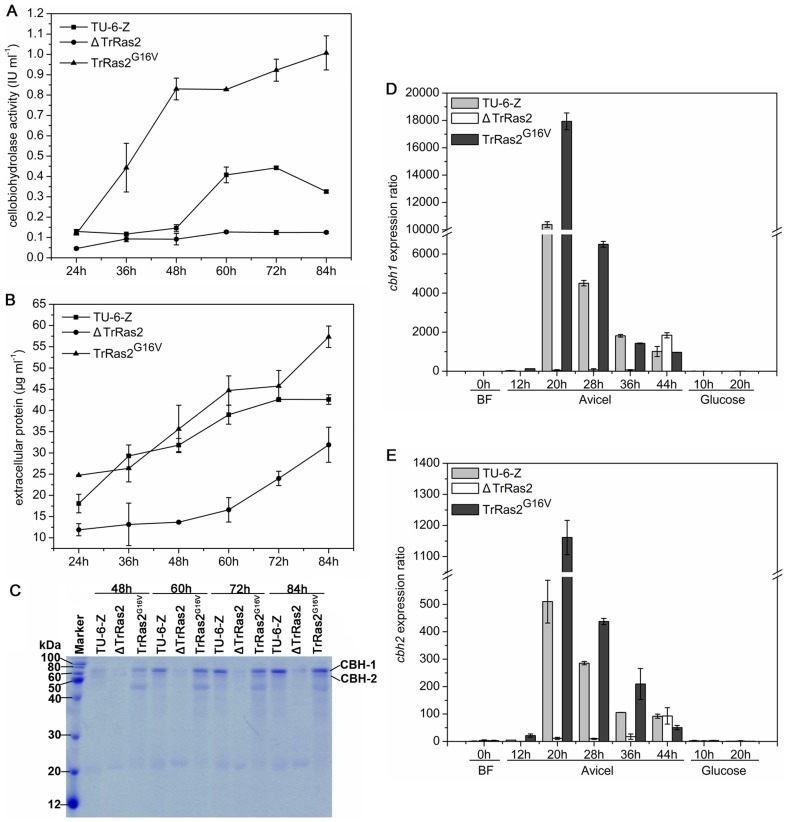
TrRas2 influences cellulase formation in *T. reesei*. Comparison of Cellobiohydrolase activity (A) and extracellular protein concentration (B) of stains TU-6-Z, Δ*TrRas2* and *PAnigpdA*-*TrRas2^G16V^* grown on 1% Avicel cellulose. (C) SDS-PAGE of secreted proteins in culture supernatants from the TU-6-Z, Δ*TrRas2* and *PAnigpdA*-*TrRas2^G16V^* strains upon growth on 1% Avicel cellulose. Transcript levels of the major cellulase genes *cbh1* (E) and *cbh2* (F) were detected just before inducing (BF, 0 h) and 12 h, 20 h, 28 h, 36 h, 44 h after the beginning of the cultivation on cellulose and 10 h, 20 h on glucose. The relative *cbh1* and *cbh2* mRNA levels were present by setting the amount of *cbh1* and *cbh2* mRNA obtained from BF as 1 respectively. Each reaction was done in triplicate.

The fact that cultivation of the Δ*TrRas2* strain on cellulose plate leads to no clear cellulolytic zone formation prompted us to detect the cellulolytic enzyme activity in the relevant mutants. The parental strain TU-6-Z, the *TrRas2* deletion strain and the dominant activated *PAnigpdA*-*TrRas2^G16V^* strain were grown to exponential phase, thus keeping the growth rate at the same level, and then equal amounts of mycelia were transferred to medium with cellulose as the sole carbon source and induced designated periods for cellulase production. Cellobiohydrolase activity (Figure 6A) and secreted protein concentration (Figure 6B) in supernatant samples of all cultivations were measured. As it can be inferred from the results, both cellobiohydrolase activity and protein concentration were strikingly reduced in the *TrRas2* deletion strain compared to the parental strain during the cultivation periods. In contrast, the *PAnigpdA*-*TrRas2^G16V^* strain secreted more extracellular protein and showed higher cellobiohydrolase activity than the parental strain (Figure 6A, B). SDS-PAGE analysis of the culture supernatants also confirmed the results mentioned above (Figure 6C). Obtained data reveal that the signal protein TrRas2 plays important roles in the cellulolytic enzyme formation in *T. reesei*.

### TrRas2 modulates expression of major cellulase genes on cellulose

Having found that the cellulase activity is greatly decreased in the Δ*TrRas2* strain, we wondered whether null mutation of TrRas2 leads to down-regulation of the cellulolytic enzyme gene expression. To address this question, the expression levels of two major cellulase genes (*cbh1* and *cbh2*) were investigated by quantitative real-time PCR (Figure 6 D, E). Similar to the previous reports [38], [39], we found that both *cbh1* and *cbh2* were greatly expressed in the parental strain upon induction by cellulose, but not by glucose. In the presence of cellulose, only marginal transcripts of both *cbh1* and *cbh2* were detectable in the *TrRas2* deletion strain at early time of cultivation. Then the transcription levels of *cbh1* and *cbh2* in the Δ*TrRas2* strain increased at the end of the fermentation (induced for 44 h) but only reached to 20% of that at 20 h in the parental strain (Figure 6D, E). These data allowed us to assume that TrRas2 may be involved in transmitting the signal from cellulose to celluase gene expression, i.e. the mutant with the constitutively activated TrRas2 should form cellulase in the absence of cellulose inducer. To test the hypothesis, the expression of *cbh1* and *cbh2* in the dominant activated *PAnigpdA*-*TrRas2^G16V^* strain on noninducing carbon source glucose was examined. However, no apparent cellulase genes expression (*cbh1* and *cbh2*) was detected in the *PAnigpdA*-*TrRas2^G16V^* strain on glucose (Figure 6D, E), indicating that constitutive activation of TrRas2 does not result in the inducer-independent celllase gene expression, i.e., TrRas2 does not directly transmit the extracellular cellulose signal to stimulate cellulase gene expression. Only *cbh1* but not *cbh2* is subjected to Cre1-dependent carbon catabolite repression [39], thus exclude the interference of glucose catabolite repression in this experiment.

Although the possibility of the direct cellulose signalling by TrRas2 was rejected, we found that cultivation of the *PAnigpdA*-*TrRas2^G16V^* strain on cellulose resulted in an early and increased (increase by 73% for *cbh1* and 128% for *cbh2* at 20 h) cellulase gene transcription compared to the parental strain (Figure 6D, E), thus indicating that this GTPase could modulate cellulase gene expression in the presence of cellulose. These data suggest that TrRas2 may sense the signals except cellulose and act upstream of the transcription regulators to modulate cellulase gene expression.

### The expression of the cellulase gene transcription factors is influenced by TrRas2

**Figure 7:**
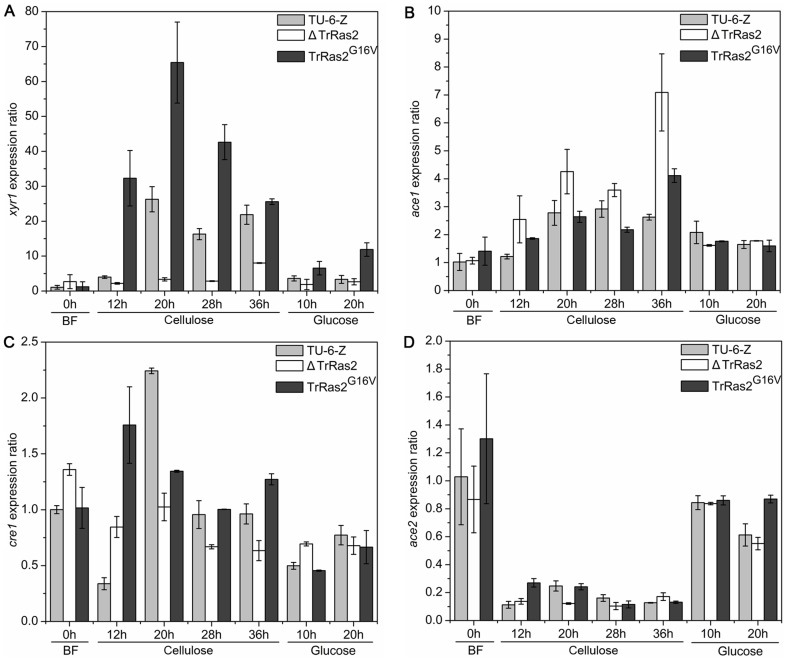
Analysis of the influence of TrRas2 on expression of the transcription factors encoding genes. Relative expression of *xyr1* (A), *ace1* (B), *cre1* (C) and *ace2* (D) from the parental strain TU-6-Z, the Δ*TrRas2* strain and the *PAnigpdA*-*TrRas2^G16V^* strain were shown. Transcripts of the target genes were detected just before inducing (BF, 0 h) and 12 h, 20 h, 28 h, 36 h, 44 h after the beginning of the cultivation on cellulose and 10 h, 20 h on glucose. The ratio obtained from BF was set to 1.

It is believed that the expression of cellulolytic gene is regulated by the cellulase transcription factors (e.g., Xyr1, Ace1, Ace2 and Cre1) in *T. reesei* [13], [14], [17], [40]. To investigate whether TrRas2 influences cellulase gene transcription through modulating the expression of these regulators, we tested the transcript abundance of *xyr1*, *ace1*, *ace2* and *cre1* in the *TrRas2* mutants. The results were shown in Figure 7. Similar to the reports of Portnoy et al. [40], we found that *xyr1* transcript accumulation in the parental strain on cellulose was higher (7.9-fold at 20 h) than that on glucose. In the presence of cellulose, *xyr1* transcript abundance in the Δ*TrRas2* strain was strikingly lower than that of the parental strain, whereas the relative expression of this gene in the *PAnigpdA*-*TrRas2^G16V^* strain was apparently higher (2.6-fold at 20 h) compared to the parental strain TU-6-Z (Figure 7A). Similar results were also found when the mutants were cultured on glucose (Figure 7A), suggesting TrRas2 positively influences the transcript level of the major regulator *xyr1* independent of inducing carbon source. Although constitutive activation of TrRas2 leads to the increase of *xyr1* transcript abundance on glucose, cellulase gene expression in *PAnigpdA*-*TrRas2^G16V^* is still undetectable on this repressing carbon source may be due to the lower levels and the inactive status of Xyr1 relative to that on cellulose. Additionally, we also found TrRas2 has a negative effect on transcript level of *ace1* but a positive influence on *cre1* transcription only in the presence of cellulose, while only has a small effect on transcript abundance of *ace2* (Figure 7B, C, D).

It has been shown that Xyr1 acts as an activator while Ace1 as a repressor in regulation of cellulase gene expression [13], [17]. The transcript patterns of *xyr1* and *ace1* in TrRas2 mutants are strictly in accordance with that of *cbh1*/*cbh2*, suggesting that TrRas2 modulates cellulase gene expression via regulation of the abundance of *xyr1*and *ace1* on cellulose. Since Ace1 possesses a negative [41] while Cre1 plays a positive role [40] in the transcription of *xyr1*, our data indicate that TrRas2 may regulate the abundance of the major transcriptional factor Xyr1 through regulating the expression level of *ace1* and *cre1*.

### Regulation of cellulase gene transcription by TrRas2 requires Xyr1

**Figure 8:**
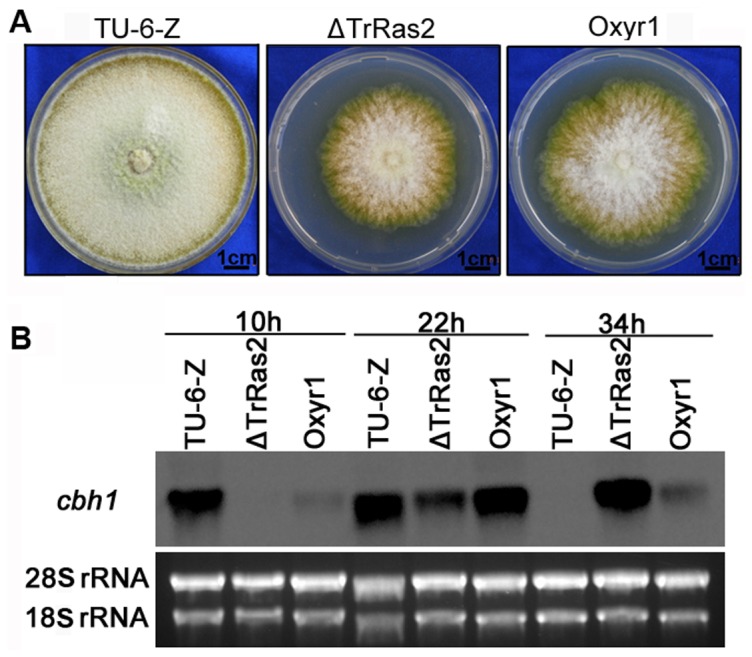
Influence of Xyr1 overexpression in the *TrRas2* deletion strain on regulation of cellulase gene transcription. (A) Phenotypes of the TU-6-Z, Δ*TrRas2* and *Oxyr1* stains upon growth on PDA plates. Strains were grown for 5 days at 30°C. Scale bar = 1 cm. (B) Northern blot analysis of *cbh1* transcription in the TU-6-Z, Δ*TrRas2* and *Oxyr1* stains. Strains were induced with 1% Avicel cellulose. A total of 2 µg RNA was loaded per lane. 28S rRNA and 18S rRNA were used as the control.

The finding that TrRas2 positively regulates the expression of the major transcriptional regulator Xyr1 raised the question of whether Xyr1 is indeed the downstream target of TrRas2 required for regulation of cellulase gene expression. In order to address this question, we constructed a mutant strain which overexpresses the *xyr1* gene under control of the constitutive *AnigpdA* promoter in the Δ*TrRas2* background (Figure S7A and B). Mutant *Oxyr1.11* was used for the following experiments. Real-time PCR analysis showed that the expression of *xyr1* in this mutant strain increased by 27% compared to TU-6-Z, while was 17.6-fold higher than that of Δ*TrRas2* (Figure S7C). This mutant did not display altered growth compared to the Δ*TrRas2* strain (Figure 8A). Cellulase gene expression strikingly decreased in the Δ*TrRas2* mutant at 10 h and 22 h, and then increased at 34 h. However, the transcription of *cbh1* could be detected at 10 h and reach to wild-type level at 22 h in the *Oxyr1* strain (Figure 8B). These data therefore indicate that overexpression of *xyr1* suppresses the cellulase gene expression defect of the Δ*TrRas2* mutant, suggesting that Xyr1 acts as one of the major downstream targets of TrRas2 during regulation of cellulase gene transcription. Overexpression of *xyr1* could not completely rescue the effect of TrRas2 deletion on *cbh1* expression, indicating that other targets may exist downstream of TrRas2 in the pathway regulating cellulase gene expression.

### TrRas1 and TrRas2 play similar roles in increasing cAMP level

**Figure 9:**
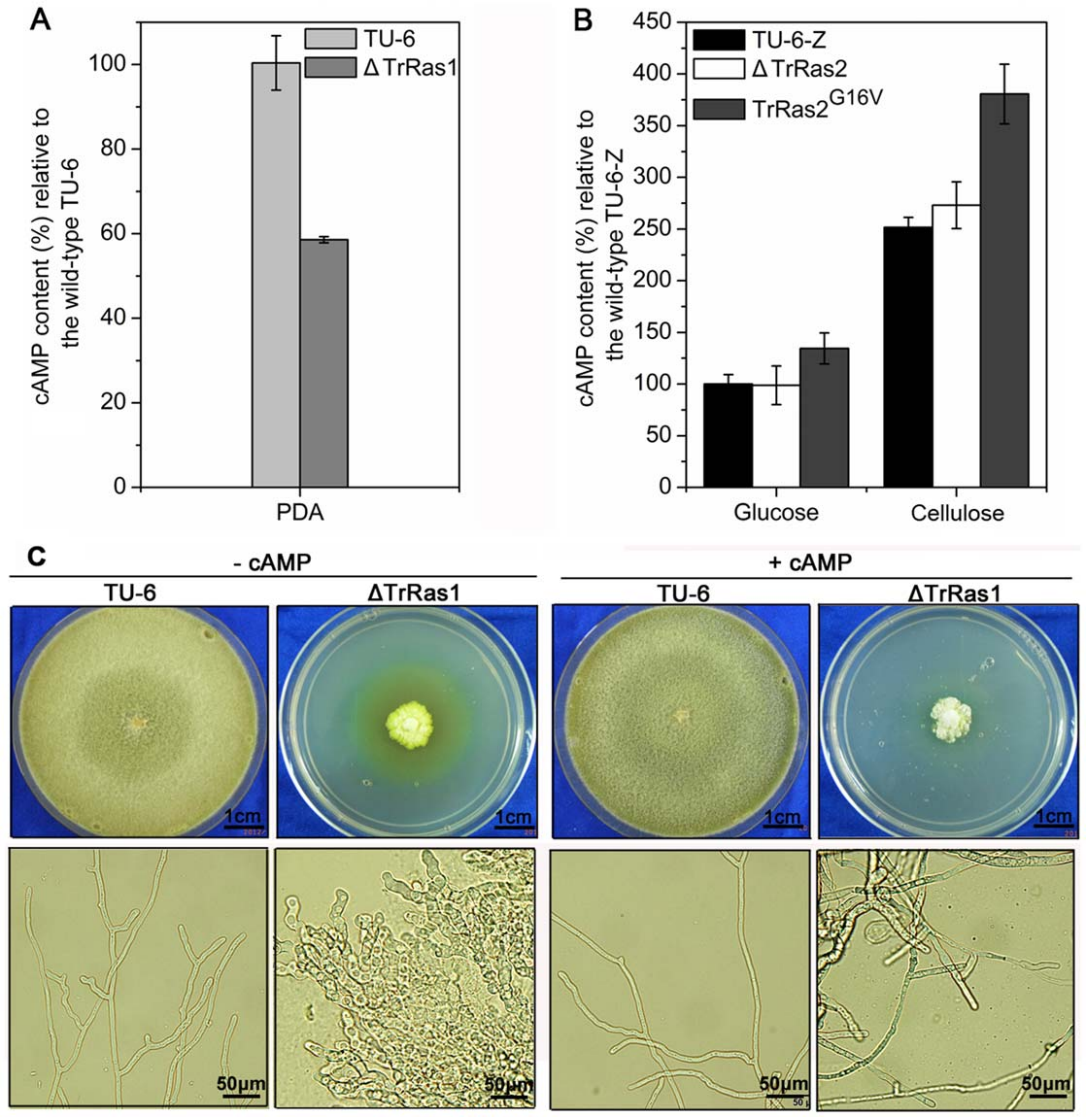
TrRas1 and TrRas2 play similar roles in increasing cAMP content. (A) The cAMP levels of TU-6 and Δ*TrRas1* on PDA plates. (B) Comparison of the cAMP levels of strains TU-6-Z, Δ*TrRas2* and *PAnigpdA*-*TrRas2^G16V^*. Cultivation of respective strains and cAMP level determination were performed as described in the methods. The cAMP content is given in relation to parental strain.(C) Phenotypes of wild-type and Δ*TrRas1* upon growth on PDA plates in the presence or absence of 2 mM cAMP in the medium. Strains were grown for 7 days at 30°C. Scale bars are shown in the figure.

In many fungi, such as *S. cerevisiae*, *C. neoformans* and *C. albicans*, Ras1/2 GTPase plays a prominent role in morphogenesis and gene expression through regulating cAMP level [32]. It has been found that cAMP pathway is involved in regulation of vegetative growth and light modulated cellulase gene expression in *T. reesei* [31]. In order to study whether Ras proteins modulate morphogenesis and cellulase gene expression via cAMP pathway in *T. reesei*, the intracellular cAMP levels in the Δ*TrRas1*, Δ*TrRas2* and *PAnigpdA*-*TrRas2^G16V^* mutants were measured. In comparison with the parental strain, the cAMP level in the Δ*TrRas1* strain decreased by 42% (Figure 9A). In contrast, the cAMP level in Δ*TrRas2* showed no changes, neither on cellulose nor on glucose (Figure 9B), thus leading us to reject the possibility that TrRas2 modulates cellulase gene expression through cAMP signalling. However, expression of the dominant activated *TrRas2^G16V^* allele resulted in 34% and 50% increase in cAMP levels on glucose and cellulose respectively (Figure 9B), which suggested that TrRas2 also plays a role in increasing cAMP concentration. An alternative explanation for no changes in cAMP level in the strain lacking TrRas2 could be that TrRas1 might be able to complement the role of TrRas2 in adjusting cAMP level. From these data, one could assume that the filamentation and sporulation defects in the Δ*TrRas1* mutant could be due to the decrease in cAMP levels. We consequently supplemented the PDA media with cAMP to investigate the growth of Δ*TrRas1* mutant. As shown in Figure 9C, the results showed that the filamentous growth and the aerial hyphae growth of the Δ*TrRas1* mutant were greatly enhanced by addition of exogenous 2 mM cAMP, although not to the wild-type level. It would be concluded that TrRas1 regulates filamentation program through cAMP signalling pathway. Similarly, RasC regulates adenylate cyclase activity through regulation of the heterotrimeric G-proteins in *Dictyostelium discoideum* [42]. However, in *A. nidulans* and *U. maydis*, RasA or Ras2 regulate morphogenesis independent of cAMP synthesis [27], [43]. In this work, our data indicate that both TrRas1 and TrRas2 may play overlapping roles in increasing cAMP concentrations while TrRas1 is more dominant than TrRas2, which provide the new evidence that Ras signalling acts through cAMP pathway to modulate morphogenesis in ascomycetous fungi.

## Discussion

Ras GTPases are critical binary switches in signalling processes that convey signals from extracellular environment to nucleus and regulate cell growth, proliferation and differentiation in eukaryotes from human to yeast [22], [23]. Ras genes are firstly identified as oncogenes in human tumors and mutations of these genes exist in almost one third of all human cancers [44]. Recently, the functions of Ras involved in fungal development, pathogenesis and gene expression have been well documented [32]. In this study, our results clearly indicate that TrRas1 and TrRas2 play both similar and distinct roles during morphogenesis and cellulase gene expression in the model cellulolytic fungus *T. reesei*. Indeed, TrRas1and TrRas2 are both involved in the regulation of polarized apical growth of hyphal cells, branch emergence and increasing cAMP level. However, TrRas1 is more dominant than TrRas2 during controlling these cellular processes. Specially, TrRas2 is also involved in the regulation of cellulase gene expression in the presence of cellulose.

Polarized apical growth is crucial for filamentation and asexual development in filamentous fungi, and Ras GTPases emerge as key regulators in this process [45], [46]. In *S. cerevisiae*, Ras1 and Ras2 are correlated with cell elongation, cell adhesion, agar invasion and the pseudohyphal growth [25], [47]. In the dimorphic pathogenic fungi, *C. albican*, CaRas1p is required for polarized growth and thereby contributes to transition from yeast-like mode of growth to filamentous growth [29]. As in *C. albican*, RasA regulates polarized growth of yeast cells and hyphae in *P. marneffei* and also regulates initiation of asexual development and branch emergence in this fungus [37]. Deletion of *A. fumigatus RasB* results in a lag in germination, a hyper-branching phenotype and a deficient colony with decreased peripheral growth and irregular borders on solid agar [48]. Kana-uchi *et al*. [49] discovered that Nc-ras2 regulates polarized growth, cell wall synthesis, aerial hyphae formation and conidiation in *N. crassa*. In *U. maydis*, signalling pathways mediated by Ras2 regulate filamentous growth while Ras1 does not influence cell morphology [27], [28]. Similarly, both TrRas1 and TrRas2 play important roles in controlling polarized apical growth in *T. reesei*. The mutant with a deletion in *TrRas1* fails to produce normal filamentous hyphae but produces a cluster of swollen and short hyphal cells, while expression of the dominant activated *TrRas2^G16V^* allele promotes polarized growth greatly. Although deletion of *TrRas2* results in a reduced colony with decreased aerial hyphae and scalloped borders, the mutant still can maintain the filamentous growth, suggesting TrRas1 and TrRas2 may be functional redundant in controlling polarized apical growth and TrRas1 is more dominant. This is similar to *S. cerevisiae* and *C. neoformans*, where Ras1 and Ras2 possess redundant cellular functions [26], [30], [50]. In addition, we also found TrRas1 and TrRas2 may play overlapping roles in increasing cAMP concentration. Despite significant efforts, a *T. reesei* strain with double deletion of *TrRas1* and *TrRas2* could not be generated suggesting such mutation may be lethal like that of *S. cerevisiae*, in which lacking Ras1 and Ras2 simultaneously is inviable [51], [52].

**Figure 10:**
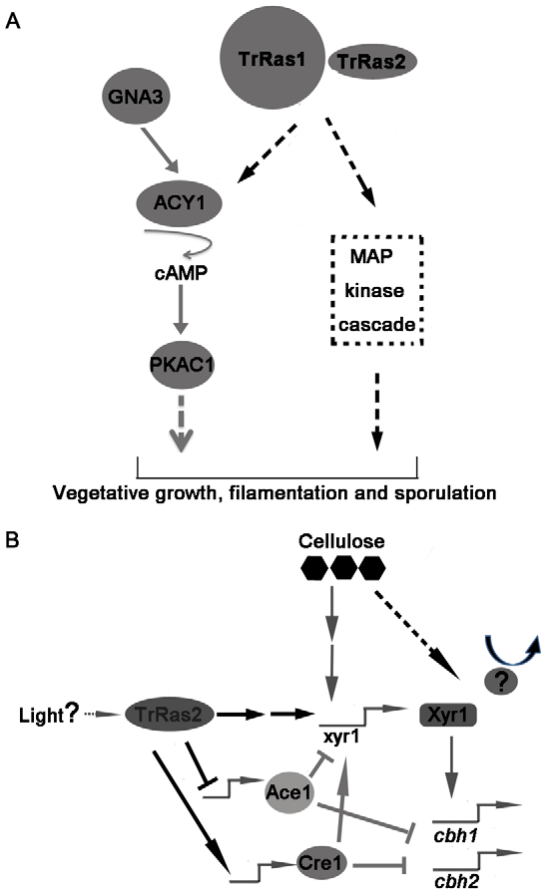
Schematic model depicting TrRas1/2 signalling in regulating morphogenesis and cellulase gene expression in *T. reesei*. The network described in this work is indicated by black lines, while regulatory relationships described previously are indicated by gray lines. Solid lines indicate genetically determined steps and dashed lines represent hypothesized steps. (A) Activation of GNA3 results in increased cAMP levels, which regulates the PKAC1 to control vegetative growth [31]. Morphogenesis is also regulated by GTPases TrRas1 and TrRas2, which probably act through cAMP-PKAC1 pathway and MAP kinase pathway to regulate filamentation, vegetative growth and sporulation. TrRas1 and TrRas2 possess similar roles in controlling these cellular processes. (B) In the presence of cellulose, TrRas2 senses extracellular signals (e.g., light) and acts through an unidentified pathway to modulate the transcript abundance of transcriptional regulators Xyr1, Ace1 and Cre1, which directly or indirectly regulate the cellulase gene expression. TrRas2 positively influences the transcription of *xyr1* and *cre1* but negatively influences *ace1* expression. Xyr1 (activator) and Ace1 (repressor) subsequently regulate cellulase genes (*cbh1* and *cbh2*) expression. Meanwhile, Ace1 negatively influences the *xyr1* expression [41], while Cre1 has a positive effect on the transcription of *xyr1* [40]. In addition, Xyr1 may also be repressed by an unknown element in the presence of glucose; while under cellulose-inducing conditions, the repressor departs from Xyr1 thus making this activator being unrepressing status.

Having found the critical roles of TrRas1 and TrRas2 in polarized apical growth, we wonder what are the signalling mechanisms that are regulated by TrRas1 and TrRas2 during filamentous growth of *T. reesei*. Schuster *et al*. [31] have found that cAMP signalling is involved in vegetative growth in *T. reesei*. In this work, our results demonstrate that deletion of *TrRas1* leads to a decrease in cAMP level and that addition of exogenous cAMP could partially rescue the filamentation defect of Δ*TrRas1*, suggesting TrRas1 may act upstream of adenylyl cyclase to regulate morphogenesis in *T. reesei*. However, the morphological defects in the Δ*TrRas1* strain are more severe than those in Δ*acy1* and Δ*pkac1*which display dramatically decreased growth rate on solid agar but normal hyphal cells and conidia formation [31]. Moreover, although deletion of *TrRas2* leads to no change in cAMP level, defects of phenotype are found in the Δ*TrRas2* mutant. These facts clearly indicate that other pathways besides cAMP signalling are regulated by TrRas1 and TrRas2 during morphogenesis. It has been shown that the maintenance of polarized growth is regulated by Cdc42 or its homologue CflA which co-localizes with actin at the hyphae apex to organize the actin cytoskeleton and that the Cdc42 or CflA activation is regulated by Ras2/RasA in *S.cerevisiae* and *P. marneffei* [37], [53]. Therefore, it is tempting to speculate that in *T. reesei* TrRas1 or TrRas2 also interacts with TrCdc42 (tre50335), the homologue of the Cdc42 in *S. cerevisiae*, to regulate the process of filamentous growth. In addition, it has been proposed in several fungi that Ras acts on a MAP kinase cascade to regulate filamentous growth through controlling cell elongation and cytokinesis (reviewed in [32]). Ras2 signals via the Cdc42/Ste20/Ste11/Ste7/Ste12 cascade to modulate cell elongation and cell adhesion, thus eventually controls the filamentous growth in *S. cerevisiae* [25], [26], [54]. Similar regulation mechanisms of Ras in controlling fungal morphogenesis via elements of MAP kinase signalling pathway are also found in *C. neoformans* and *C. albicans* [29], [36]. Ras2 of *U. maydis* acts opposed to cAMP-PKA pathways, but through a MAP kinase pathway to regulate morphogenesis [27]. These facts promote us to analyze whether the analogous pathways controlling filamentous growth exist in *T. reesei* and depend upon TrRas1 and TrRas2. By analogy with *S. cerevisiae*, we found homologues of the MAP kinase signalling components involved in filamentous growth, TrSte20 (tre104364), TrSte11 (tre4945), TrSte7 (tre75872) and TrSte12 (tre36543), are existed in *T. reesei* genome. Consequently, we proposed the model of TrRas1 and TrRas2 signalling (as illustrated in Figure 10A) that may be involved in filamentation, vegetative growth and asexual development in *T. reesei*. In fact, the relationships between TrRas1 and TrRas2 signalling and MAP kinase cascade in controlling filamentous growth are being studied in our lab.

The mutant Δ*TrRas1* still can degrade cellulose to form a clear zone on cellulose plate despite it shows a severe growth defect, while deletion of *TrRas2* interrupts the cellulose degrading on the plate. Therefore, we focus our study on exploring the influence of TrRas2 on cellulase gene expression. Deletion of *TrRas2* leads to a great decrease in the transcription of major cellulase genes, suggesting that TrRas2 is involved in cellulase gene expression. However, constitutive activation of TrRas2 does not lead to cellulase gene transcript independent of inducer, indicating that TrRas2 is not directly involved in transmitting the signal from cellulose to cellulase gene expression. It has been shown that light acts through signalling pathway involving heterotrimeric G-proteins/cAMP/PKC1 to modulate cellulase gene transcription in *T. reesei* [19], [31], [55]. In silico analysis of 2 kb upstream of the *TrRas2* translational start codon reveals two single EUM1-binding motifs (EUM1, **e**nvoy **u**pstream **m**otif 1) [55] (positions −1228 to 1223 and positions −1748 and −1743) which have been described to occur in genes regulated by light. Thus, it seems that TrRas2 signalling may transmit the light or other signals to modulate cellulase gene expression.

It has been discovered that cAMP signalling is involved in regulation of cellulase gene expression [19], [31]. Moreover, the adenylate cyclase, which produces the cAMP as a secondary messenger in many cellular functions, is activated by Ras GTPase in plenty of fungi (reviewed in [32]). Based on these facts, we propose the hypothesis that TrRas2 may modulate the cellulase gene expression through cAMP signalling pathway. However, no alteration in the cAMP level on cellulose or glucose is detected in the mutant with a deletion for *TrRas2*, indicating that influence of TrRas2 on cellulase gene transcription is independent of cAMP signalling pathway.

Xyr1 has been demonstrated as a central transcriptional regulator that controls xylanolytic as well as cellulolytic enzyme genes expression in *T. reesei* [13]. The transcription of almost all cellulase genes is significantly impaired or reduced in the *xyr1*-konckout strain under inducing condition [56]. In this work, *xyr1* transcription is greatly decreased in the Δ*TrRas2* mutant while increased in the *TrRas2^G16V^* transformants, indicating that TrRas2 may modulate cellulase gene expression by regulating the abundance of Xyr1. The fact that overexpression of *xyr1* in the Δ*TrRas2* background could rescue the defect of cellulase gene expression further confirmed that Xyr1 acts as one of the major downstream targets of TrRas2 during regulation of cellulase gene transcription. In addition, we also find that TrRas2 has a negative effect on *ace1* transcript while a positive effect on *cre1* expression in the presence of cellulose. Since Ace1 is shown as a repressor in the expression of the *xyr1* [41] and Cre1 is necessary for the full induction of *xyr1* transcript on cellulose [40], our data suggest the possibility that TrRas2 also modulate the abundance of Xyr1 via modulating transcription of *ace1* and *cre1*. Taken together, it is convincible that TrRas2 senses extracellular signals (e.g., light) and acts through an unidentified pathway to modulate the expression of transcriptional regulators which further regulate the cellulase gene transcription (Figure 10B).

In conclusion, our results show that TrRas1 and TrRas2 play similar and distinct roles in morphogenesis in the model cellulolytic fungus *T. reesei*. Moreover, signalling pathways, but not the cAMP signalling, mediated by TrRas2 are involved in modulating cellulase gene expression through regulating the transcription of cellulase gene transcriptional regulators. Identification of the downstream components of TrRas1 and TrRas2 during filamentation and cellulase gene expression will be a major challenge for future studies. In addition, it will be fascinating to study the true extracellular signals transmitted by TrRas2 during modulation of cellulase production.

## Materials and Methods

### Strains, cultural conditions and microscopy

*T. reesei* strains QM9414 (ATCC 26921), TU-6 (ATCC MYA-256; uridine auxotroph [57], *pyr4* ^−^), TU-6-Z (the strain transformed with *pyrG* cassette from pAB4-1 [35], *pyrG* ^+^), Δ*TrRas1* (Δ*TrRas1*::*ptrA* ^+^), *cbh1-TrRas1* [*cbh1(p)*-*TrRas1*::*ptrA* ^+^], Δ*TrRas2* (Δ*TrRas2*::*pyrG* ^+^), Re*TrRas2* (Re*TrRas2*::*ptrA^+^,* Δ*TrRas2*::*pyrG* ^+^), *PAnigpdA*-*TrRas2^G16V^* [*gpdA(p)*-*TrRas2^G16V^*::*pyrG* ^+^] and *Oxyr1* [*gpdA(p)*-*xyr1*::*ptrA* ^+^, Δ*TrRas2*::*pyrG* ^+^] were used throughout this study. All the strains were maintained on potato dextrose agar (PDA) plates, or supplemented with 10 mmol L^−1^ uridine when necessary (for strains TU-6, Δ*TrRas1* and *cbh1-TrRas1*). *Escherichia coli* DH5α was used for cloning of constructs and cultured in LB broth supplemented with appropriate antibiotics at 37°C.

To analyze the transcript levels of *TrRas1* and *TrRas2* on different carbon sources, replacement experiments were designed. Pregrown mycelia of 0.6 g were transferred to 150 ml minimal medium containing different carbon sources (e.g. 2% glucose, 2% glycerol and 1% Avicel cellulose) and grown for additional 6 h, 24 h and 48 h. To detect the expression level of *TrRas2* in Δ*TrRas1* (and vice versa), equivalent squares of agar with growing strains, TU-6-Z, Δ*TrRas1* and Δ*TrRas2*, were grown for 12 h, 24 h and 48 h at 30°C and 200 rpm in 150 ml liquid glucose minimal medium. Then mycelia were harvested and used for RNA extraction.

For assays of growth on different carbon sources, equivalent squares of agar with growing strains were inoculated on plates with minimal medium supplemented with 1% (w/v) of the corresponding carbon source and 2% agar for 4 days at 30°C.

Strains TU-6-Z, Δ*TrRas2* and *PAnigpdA*-*TrRas2^G16V^* were cultured in liquid glucose minimal medium or on PDA plates to determine growth rate according to the method of Aro *et al*. [14].

For induction experiments [56], equivalent squares of agar with growing strains, TU-6-Z, *PAnigpdA*-*TrRas2^G16V^* and Δ*TrRas2*, were grown for 30 h, 36 h and 48 h at 30°C and 200 rpm in 200 ml liquid glucose minimal medium (LMM). Mycelia were collected by filtration, washed twice with sterilized water, and equal amounts of mycelia (1 g) were transferred to 200 ml minimal medium with 1% Avicel cellulose (w/v) as the inducer or 1% glucose (w/v) as the control. For RNA extraction, induction was performed for 12 h, 20 h, 28 h, 36 h and 44 h on Avicel cellulose or 10 h and 20 h on glucose at 30°C and 200 rpm. For cellulase activity assay, culture medium samples were collected at 24 h, 36 h, 48 h, 60 h, 72 h and 84 h after induction. For detection of *cbh1* expression in the *Oxyr1* strain, RNA was extracted after induced for 10 h, 22 h, and 34 h.

Strains TU-6, Δ*TrRas1* and *cbh1-TrRas1* were inoculated on plates with PDA or minimal medium containing 1% (w/v) Avicel cellulose as the sole carbon source for 5 days at 30°C to determine the morphological phenotypes. Strains TU-6-Z, Δ*TrRas2*, *PAnigpdA*-*TrRas2^G16V^* and *Oxyr1* were grown on PDA plates or liquid minimal medium with 2% glucose (w/v) as the carbon source for morphological phenotype assays. Photographs of colonies were taken with a Samsung Digimax S500 camera. Microscopic images were captured on Nikon eclipse 80i light microscope (Nikon, Japan).

### Molecular techniques

*T. reesei* genomic DNA isolation was carried out as described previously [58]. PCR experiments were performed with standard protocols using a T1 Thermocycler (Biometra, Gottingen, Germany) unless otherwise indicated. Prime design was carried out using the primer premier 5.00 software (PREMIER Biosoft). DNA fragments were purified using Gel Extraction Kit (Omega, USA). Oligonucleotides synthesis and DNA sequencing were performed at Sangon Inc. (Shanghai, China). Oligonucleotides used in this study are listed in Table S1 in supplementary materials. Using chromosomal DNA and cDNA of *T. reesei* as the templates, *TrRas1* or *TrRas2* genes were amplified with primer pairs Ras1-RT-S/Ras1-RT-A or Ras2-RT-S/Ras2-RT-A respectively. Then the fragments were cloned into pMD18-T (Takara, Japan) to sequence the wild-type *TrRas1* and *TrRas2* genes. The related DNA sequence data have been deposited in GenBank. Standard procedures were applied for other molecular manipulations [59]. Multiple alignments of protein sequences were performed with ClustalW2 (http://www.ebi.ac.uk/Tools/msa/clustalw2/). Phylogenetic analysis was inferred using the Neighbor-joining method and the software MEGA4.0. The *T. reesei* QM6a DNA sequence and protein predictions of the *T. reesei* genome database ver2.0 were used in this study (http://genome.jgi-psf.org/Trire2/Trire2.home.html).

### Construction of *T. reesei* mutants

Fusion PCR and gene targeting were used for producing the mutants of this study as described previously [58], [60]. To construct *TrRas1* deletion strain (Δ*TrRas1*), a deletion construct Δ*TrRas1*::*ptrA* ^+^ was designed as following: firstly, 5′ *TrRas1* flanking region, 3′ *TrRas1* flanking region and a pyrithiamine resistance cassette *ptrA* were amplified using primer pairs Ras1-5-S/Ras1-5-A, Ras1-3-S/Ras1-3-A and ptrA-S/ptrA-A respectively. Then these PCR fragments were mixed and used as the template to obtain the deletion cassette by using the nest primer pair Ras1-nest-S/Ras1-nest-A. Δ*TrRas1*::*ptrA* ^+^ was used to transform *T. reesei* TU-6. Transformant with a deletion at the *TrRas1* locus was firstly identified by PCR using primer pair YΔRas1-S/YΔRas1-A, which resulted in a 2.4 kb fragment in the transformant, whereas a 1.7 kb fragment in the wild-type strain. Similarly, another deletion cassette, Δ*TrRas2*::*pyrG* ^+^, was constructed to obtain null mutation at the *TrRas2* locus with *pyrG* as the selective marker. Δ*TrRas2*::*pyrG* ^+^ was obtained from fusion PCR of three fragments, 5′ *TrRas2* flanking region, 3′ *TrRas2* flanking region and a *pyrG* cassette generated by using primer pairs Ras2-5-S/Ras2-5-A, Ras2-3-S/Ras2-3-A and pyrG-S/pyrG-A respectively, via using nest primer pair Ras2-nest-S/Ras2-nest-A. PCR identification of Δ*TrRas2* mutant using primer pair YΔRas2-S/YΔRas2-A yielded a 1.7 kb fragment when the wild-type *TrRas2* was present, whereas a 3.1 kb fragment in the mutant. RT-PCR analysis was performed to determine the absence of the *TrRas1* and *TrRas2* mRNA using primer pairs Ras1-RT-S/Ras1-RT-A and Ras2-RT-S/Ras2-RT-A respectively. The cassette, Re*TrRas2*::*ptrA^+^*, used for *TrRas2* retransformation was constructed by fusion PCR, and then was retransformed into Δ*TrRas2*. The *ptrA* and *TrRas2* expression cassette were amplified using primer pairs ptrA-S/ptrA-A and Rras2-S/Ras2-nest-A respectively, then Re*TrRas2*::*ptrA^+^* was obtained through fusion of these two fragments by using primer pair Rras2-nest-S/Rras2-nest-A. Re*TrRas2* transformant was analyzed by PCR using primer pair Rras2-nest-S/Rras2-nest-A, which produced a 5.3 kb fragment in the transformant. The regain of *TrRas2* transcript was detected by RT-PCR using primer pair Ras2-RT-S/Ras2-RT-A. To replace the native promoter of *TrRas1* with the inducible *cbh1* promoter, the cassette *cbh1(p)*-*TrRas1*::*ptrA* ^+^ was introduced into *T. reesei* TU-6. The cassette *cbh1(p)*-*TrRas1*::*ptrA* ^+^ consisted of 5′ flanking region of *TrRas1*promoter, selective marker *ptrA*, *cbh1* promoter and 3′ flanking region of *TrRas1*promoter, which were obtained using primer pairs Ras1-5-S/TPRas1-5-A, APcbh1-nest-S/ptrA-A, Pcbh1-S/Pcbh1-A and TPRas1-3-S/TPRas1-3-A respectively, was generated by fusion PCR using primer pair Ras1-5-S/TPRas1-nest-A. Transformant with a successful replacement of *TrRas1*promoter was firstly identified by PCR using primer pair YTPRas1-S/YTPRas1-A, which yield a 1.8 kb product in the mutant. *Cbh1-TrRas1* mutant was also identified by using primer pair Ras1-5-S/YΔRas1-A, which resulted in a 5.4 kb fragment in the replacement strain, whereas a 3.2 kb fragment in the wild-type strain.

For expression of a constitutively activated version of TrRas2^G16V^, which had a single amino acid mutation at codon 16 (glycine to valine), *PAnigpdA-TrRas2^G16V^* (*gpdA(p)*-*TrRas2^G16V^*::*pyrG* ^+^) strain was constructed. Primer pairs Ras2-nest-S/O5Ras2-A and O3Ras2-S/Ras2-nest-A containing the mutation to be introduced into the wild-type template DNA were used to amplify the 5′ portion and 3′ portion of *TrRas2* respectively. Then these two fragments were fused with primer pair Ras2-RT-S/Ras2-nest-A and cloned into pMD18-T to make pTrRas2^G16V^. Coding region of *TrRas2^G16V^* was completely sequenced to ensure that only the desired mutations had been introduced. The *AnigpdA* promoter generated from pAN7-1 using primer pair PgpdA-S/PgpdA-A was digested with *Eco*RI and ligated to the *Eco*RI-*Sma*I sites of pTrRas2^G16V^ to make pPTrRas2^G16V^. A 2.7 kb pyrG fragment generated from pAB4-1 using primer pair pyrG-*Eco*RI-S/pyrG-*Eco*RI-A was inserted into pPTrRas2^G16V^ digested with *Eco*RI to generate pORas2. The pORas2 was used for transformation of *T .reesei* TU-6. Transformant with an integration of pORas2 was firstly tested with primer pair YORas2-S/YORas2-A (YORas2-S binds within the *AnigpdA* promoter region and YORas2-A binds within the TrRas2^G16V^ codon region), which resulted in a 2.0 kb product only in the transformant.

For overexpression of *xyr1* in the Δ*TrRas2* mutant, the *Oxyr1*::*ptrA* ^+^ cassette was constructed as following. Firstly, a 2.1 kb pyrithiamine resistance cassette *ptrA*, a 1.2 kb *AnigpdA* promoter from pAN7-1 and a 3.8 kb wild-type *xyr1* gene were amplified using primer pairs ptrA-S/ptrA-A, Oxyr1-001/PgpdA-A and Oxyr1-002/Oxyr1-003 respectively. Then the *Oxyr1*::*ptrA* ^+^ cassette was obtained through fusion of these three fragments by using primer pair ptrA-S/Oxyr1-004. The *Oxyr1*::*ptrA* ^+^ cassette was applied to transform the Δ*TrRas2* strain and pyrithiamine resistant *Oxyr1* transformants were chosen for the Southern analysis.

### RNA extraction and quantitative real-time reverse transcription PCR

For RNA extraction, mycelia were harvested by filtration, homogenized in Mini-BeadBeater (Biospec, USA) with 0.5 mm Zirconium/Silica beads at 4°C, and then total RNA were isolated with Trizol reagent kit (Life Technologies, USA).

Synthesis of cDNA from total RNA was performed using PrimeScript RT reagent Kit (Takara, Japan) as the manufacturer's instructions. Real-time PCRs were carried out in a LightCycler 480 System (Roche Diagnostics, Germany). All PCRs were performed in triplicate in 20 ul reaction mixtures containing 1×SYBR *Premix Ex Taq*™, 0.2 µmol L^−1^ forward primer, 0.2 µmol L^−1^ reverse primer, and 2 ul cDNA template (100-fold diluted) using the SYBR *Premix Ex Taq*™ (Tli RNaseH Plus) kit (Takara, Japan). Real-time PCR protocols were as following: 1 min initial denaturation at 95°C, followed by 40 cycles of 5 s at 95°C, 20 s at 60°C. Melting curve analysis with a temperature gradient of 0.1°C s^−1^ from 65°C to 95°C was performed. LightCycler480 software 1.5.0 was used to calculate Ct value. Transcript levels of target genes were normalized against the level of actin gene with ddCt method [61].

### Northern hybridization analysis

Northern blotting analysis was performed using DIG Northern Starter Kit (Roche Diagnostics, Germany) according to the manufacturer's instruction. The *cbh1* fragment obtained from PCR using primer pair NT7-CBH1-S/NT7-CBH1-A was used as the template for labeling RNA probe with digoxigenein.

### Southern hybridization analysis

Transformants constructed in this study were confirmed by Southern blotting analysis using DIG Easy Hyb kit (Roche Diagnostics, Germany) according to the manufacturer's instruction. Probe A, B and C generated from PCR using primer pairs Ras1-probe-s/Ras1-5-A, Ras1-5-S/Ras1-5-A and Ras2-Nest-S/Ras2-5-A were used for hybridization for Δ*TrRas1*, *cbh1-TrRas1* and Δ*TrRas2* respectively. Hybridization of *Bam*HI-digested genomic DNA using probe A yielded a 0.9 kb fragment in a strain with the Δ*TrRas1* allele, whereas a 10 kb fragment was observed in the wild-type strain. Hybridization of *Bal*I or ApaI-digested genomic DNA with probe B resulted in a 3.5 kb or 2.0 kb fragment in the wild-type strain while a 3.7 kb or 2.4 kb fragment in the *cbh1-TrRas1* mutant respectively. Similarly, Southern hybridization of *Bam*HI-digested genomic DNA with probe C yielded a 2.2 kb fragment in the wild-type strain and a 3.3 kb fragment in the Δ*TrRas2* strain. In addition, the integration of the *PAnigpdA*-*TrRas2^G16V^* allele into the *T. reesei* genome was analyzed by Southern hybridization using *Xho*I-digested genomic DNA with PCR probe D obtained using primer pair Ras2G16V-probe-S/Ras2G16V-probe-A, which yielded a 3.0 kb band in both the wild-type and *PAnigpdA*-*TrRas2^G16V^* strain and an additional 1.9 kb band in the *PAnigpdA*-*TrRas2^G16V^* mutant. The integration and copy number of *PAnigpdA*-*TrRas2^G16V^* allele in the genome of *T. reesei* were also analyzed by hybridization of *Sac*I-digested genomic DNA with PCR probe E generated using primer pair Ras2-inner-S/Ras2-inner-A, an additional 5.0 kb band in the mutant and the presence of the 3.0 kb wild-type band in both wild-type and mutant confirmed that the *PAnigpdA*-*TrRas2^G16V^* cassette integrated ectopically. Integration and copy number of *Oxyr1*::*ptrA* ^+^ cassette in the *Oxyr1* transformants were analyzed by Southern hybridization using *Hind*III, which located in the 3′ part of *xyr1*, thereby showing the presence of the cassette by the presence of an additional band longer than 0.87 kb. The presence of the 1.68 kb wild-type band in both Δ*TrRas2* and *Oxyr1* strains confirmed the ectopically integration of the cassette. Graphical representations of the relevant gene loci in this work were shown in Figure S1, S2, S3, S4, and S7.

### Enzyme activity assay and protein measurement

Cellobiohydrolase activity was measured as described by Murray *et al*. [62] with p-nitrophenyl-β-D-cellobioside as substrate. One unit of activity was defined as the amount of enzyme required to release one microgram of p-nitrophenyl per minute under the defined assay conditions. Protein concentration was determined using the Bio-Rad Protein Assay kit (BIO-RAD, USA).

### Intracellular cAMP assay

To measure the intracellular cAMP concentration, TU-6 and Δ*TrRas1* were grown on PDA plates for 3 days, and strains TU-6-Z, Δ*TrRas2* and *TrRas2^G16V^* were grown on liquid minimal medium with glucose or Avicel cellulose as the carbon source for 30 h, 36 h and 48 h respectively at 30°C and 200 rpm. Mycelia were collected, grounded into powder with liquid nitrogen, homogenized in 10 vols 0.1 mol L^−1^ HCl and centrifuged at 700 *g* and room temperature for 10 minutes. The cAMP level was measured using the Direct cAMP enzyme immunoassay kit (Sigma-Aldrich, St. Louis, MO) according to the manufacturer's instructions. The intracellular cAMP concentration was expressed as the relative amount compared to the wild-type strain.

## Supporting Information

Figure S1 **Deletion of** ***T. reesei TrRas1*** **.** (A) Graphical representation of the *TrRas1* genomic locus from the wild-type strain TU-6 and Δ*TrRas1*. Primer pairs and relative positions of the *Bam*HI restriction sites are given. Probe A used for Southern analysis is shown as red box. (B) PCR analysis of the transformants showed that the *TrRas1* gene had been deleted successfully. Ectopic integration of the deletion cassettes lead to an additional hybridizing fragment in ΔTrRas1.1. (C) Southern blot of the chromosome digested with *Bam*HI confirmed the deletion of *TrRas1* gene. (D) RT-PCR analysis indicated the loss of *TrRas1* mRNA in the mutant.
(TIF)

Figure S2 **Deletion and retransformation of** ***T. reesei TrRas2*** **.** (A) Schematic representation of the genomic organization of the *TrRas2* locus in TU-6, Δ*TrRas2* and Re*TrRas2* strains. Primer pairs and relative positions of the *Bam*HI restriction sites are given. Probe B used for Southern analysis is shown as red box. (B) PCR analysis showed the successful deletion of *TrRas2* gene in the mutants. (C) Southern blot of the chromosome digested with *Bam*HI confirmed the deletion of *TrRas2* gene. (D) RT-PCR analysis showed the absence and regain of the transcript of *TrRas1* in the relevant mutants. (E) PCR analysis indicated the regain of *TrRas2* expression cassette in the ReTrRas2 transformants.
(TIF)

Figure S3 **Replacement of the native** ***TrRas1*** **promoter with the regulatable** ***cbh1*** **promoter.** (A) Schematic representation of the genomic organization of the *TrRas1* promoter locus in TU-6 and *cbh1*-*TrRas1* mutant. Primer pairs and relative positions of the *Apa*I and *Bal*I restriction sites are given. Probe C used for Southern analysis is shown as black box. (B)(C) PCR analysis showed that the native *TrRas1* promoter had been replaced by *cbh1* promoter successfully and that there was no ectopic integration of the replacement casstte. (D) Southern blot of the chromosome digested with *Apa*I or *Bal*I confirmed the replacement of the native *TrRas1* promoter in the mutant.
(TIF)

Figure S4 **Construction of** ***PAnigpdA*** **-** ***TrRas2^G16V^*** **strains expressing the dominant activated** ***TrRas2*** **allele.** (A) Graphical representation of the *TrRas2* genomic locus from the wild-type strain TU-6 and *PAnigpdA*-*TrRas2^G16V^* strains. Primer pairs and relative positions of the *Sac*I and *Xho*I restriction sites are given. Probes D and E used for Southern analysis are shown as red boxes. (B) PCR analysis showed the successful integration of the pORas2 plasmid into the genome of TU-6. (C) Southern blot of the chromosome digested with *Xho*I confirmed the integration of the *gpdA(p)*-*TrRas2^G16V^* cassette into the genome of TU-6. (D) Southern blot of the chromosome digested with *Sac*I confirmed that the *gpdA(p)*-*TrRas2^G16V^* cassette integrated ectopically. (E) Quantitative real-time PCR analysis showed that the *TrRas2* mRNA levels in the *PAnigpdA*-*TrRas2^G16V^* strain was significantly increased when compared to that of the wild-type strain. The relative mRNA levels were presented by setting the amount of *TrRas2* mRNA at 9 h in wild-type strain as 1. Actin gene was used as the reference.
(TIF)

Figure S5 **Radial growth and biomass of the TU-6-Z, Δ** ***TrRas2*** **and** ***PAnigpdA-TrRas2^G16V^*** **strains.** (A) Radial growth rates of the *TrRas2* mutants. Equivalent squares of agar with growing strains were cultured on PDA plates at 30°C. (B) Biomass accumulation in LMM at 200 rpm and 30°C with equivalent squares of agar with growing relevant strains as the inoculation.
(TIF)

Figure S6 **Construction of the dominant active** ***TrRas2^G16V^*** **mutant under the control of its own promoter.** (A) Schematic representation of the genomic organization of the *TrRas2* locus in the TU-6 and *TrRas2^G16V^* strains. Relative positions of the *Bam*I restriction sites are given. Probe G used for Southern analysis is shown as red box. (B) Southern blot of the chromosome digested with *Bam*I confirmed that the *TrRas2^G16V^::pyrG^+^* cassette integrated ectopically. (C) Growth phenotypes of strains TU-6-Z and *TrRas2^G16V^* on PDA plates. One *TrRas2^G16V^* mutant was chosen as the representative. Scale bar = 1 cm.
(TIF)

Figure S7 **Overexpression of Xyr1 in the Δ** ***TrRas2*** **mutant.** (A) Schematic representation of the genomic organization of the *xyr1* locus in the Δ*TrRas2* and *Oxyr1* strains. Relative positions of the *Hind*III restriction sites are given. Probe F used for Southern analysis is shown as red box. (B) Southern blot of the chromosome digested with *Hind*III. The 1.68 kb wild-type band is present in all the strains. An additional band longer than 0.87 kb indicates the presence of *Oxyr1*::*ptrA* ^+^ cassette. (C) Quantitative real-time PCR analysis of *xyr1* in theTU-6-Z, Δ*TrRas2* and *Oxyr1* strains. Strains were induced on 1% Avicel cellulose for 22 h. The relative mRNA levels were presented by setting the amount of *xyr1* mRNA in Δ*TrRas2* as 1. Actin gene was used as the reference.
(TIF)

Text S1 **Expression of the dominant active** ***TrRas2^G16V^*** **allele under control of its own promoter.**
(DOCX)

Table S1 **Primers used in this study.**
(DOCX)
