# Supplementary figures and images for: Ras GTPases Modulate Morphogenesis, Sporulation and Cellulase Gene Expression in the Cellulolytic Fungus Trichoderma reesei
Source: PLoS One. 2012 Nov 9;7(11):e48786. doi: 10.1371/journal.pone.0048786 (PMC3494722; doi:10.1371/journal.pone.0048786)

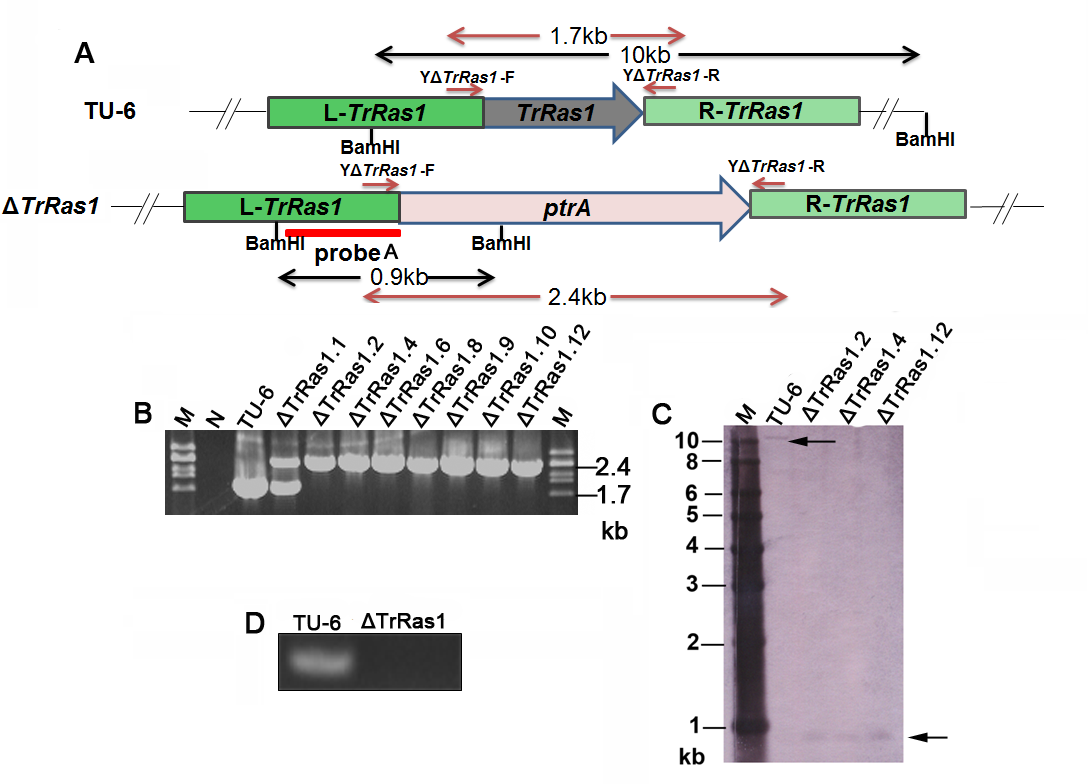

Supplement: Figure S1 — Deletion of T. reesei TrRas1 . (A) Graphical representation of the TrRas1 genomic locus from the wild-type strain TU-6 and ΔTrRas1. Primer pairs and relative positions of the BamHI restriction sites are given. Probe A used for Southern analysis is shown as red box. (B) PCR analysis of the transformants showed that the TrRas1 gene had been deleted successfully. Ectopic integration of the deletion cassettes lead to an additional hybridizing fragment in ΔTrRas1.1. (C) Southern blot of the chromosome digested with BamHI confirmed the deletion of TrRas1 gene. (D) RT-PCR analysis indicated the loss of TrRas1 mRNA in the mutant. (TIF) [file pone.0048786.s001.tif]

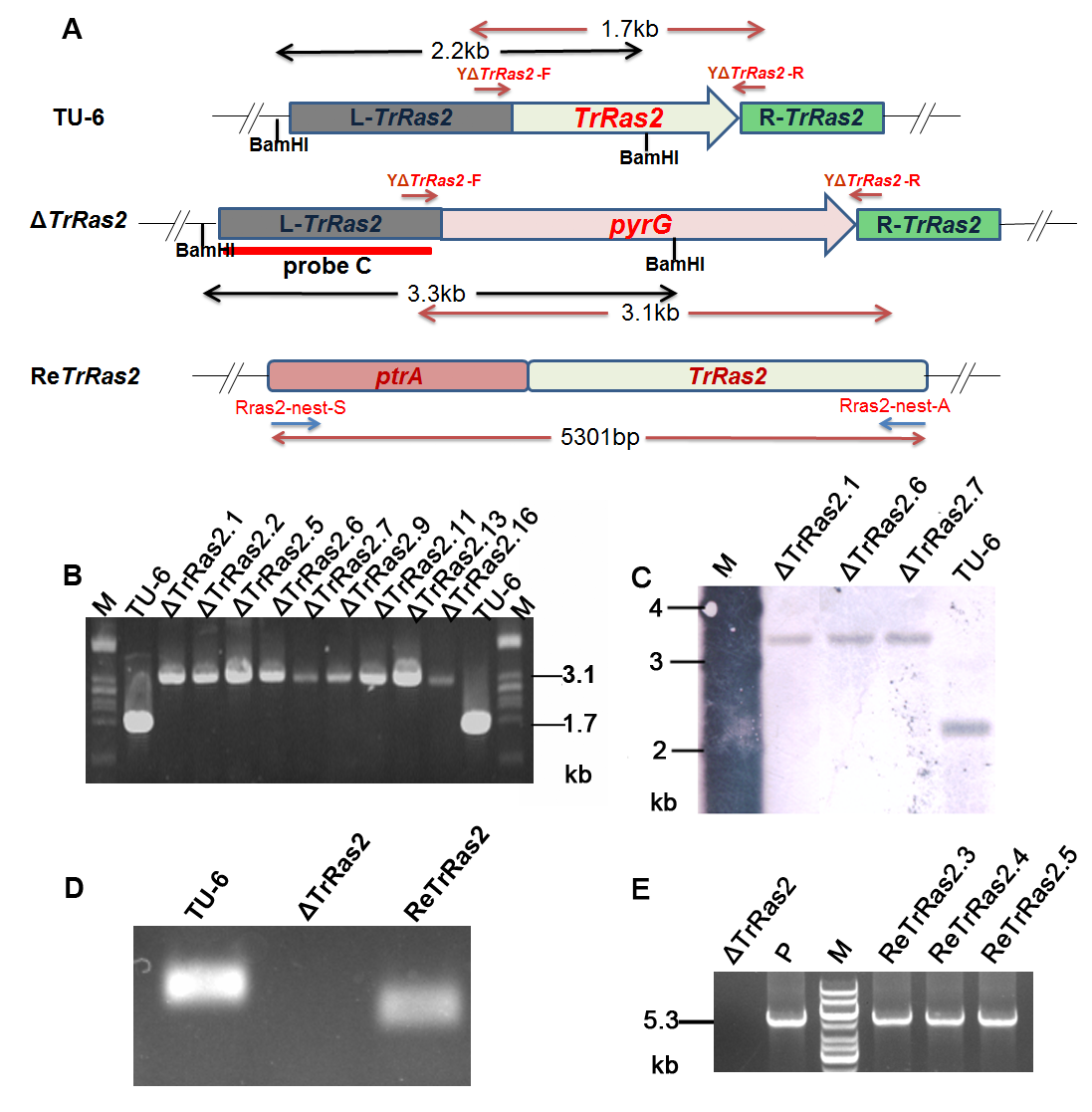

Supplement: Figure S2 — Deletion and retransformation of T. reesei TrRas2 . (A) Schematic representation of the genomic organization of the TrRas2 locus in TU-6, ΔTrRas2 and ReTrRas2 strains. Primer pairs and relative positions of the BamHI restriction sites are given. Probe B used for Southern analysis is shown as red box. (B) PCR analysis showed the successful deletion of TrRas2 gene in the mutants. (C) Southern blot of the chromosome digested with BamHI confirmed the deletion of TrRas2 gene. (D) RT-PCR analysis showed the absence and regain of the transcript of TrRas1 in the relevant mutants. (E) PCR analysis indicated the regain of TrRas2 expression cassette in the ReTrRas2 transformants. (TIF) [file pone.0048786.s002.tif]

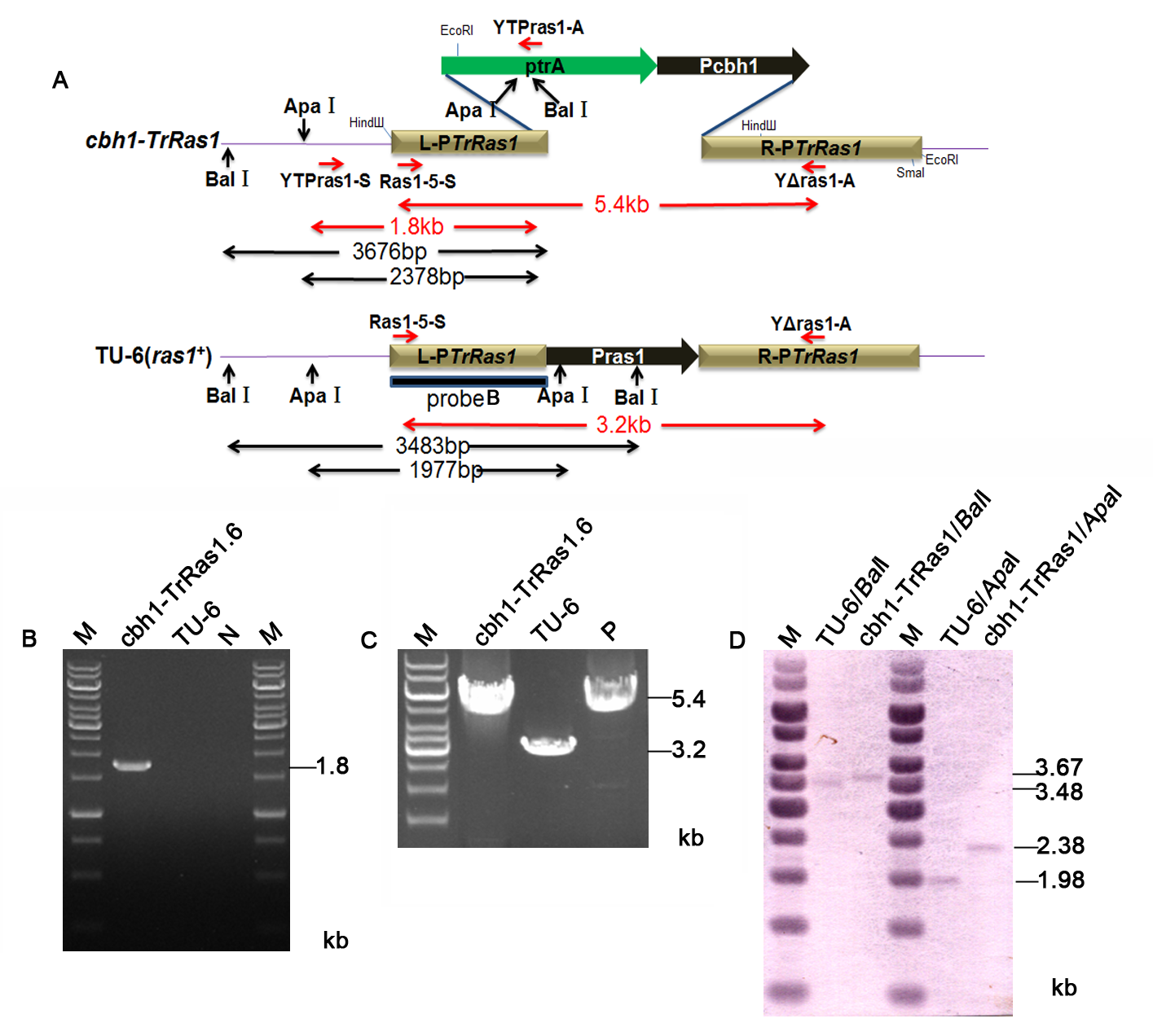

Supplement: Figure S3 — Replacement of the native TrRas1 promoter with the regulatable cbh1 promoter. (A) Schematic representation of the genomic organization of the TrRas1 promoter locus in TU-6 and cbh1-TrRas1 mutant. Primer pairs and relative positions of the ApaI and BalI restriction sites are given. Probe C used for Southern analysis is shown as black box. (B)(C) PCR analysis showed that the native TrRas1 promoter had been replaced by cbh1 promoter successfully and that there was no ectopic integration of the replacement casstte. (D) Southern blot of the chromosome digested with ApaI or BalI confirmed the replacement of the native TrRas1 promoter in the mutant. (TIF) [file pone.0048786.s003.tif]

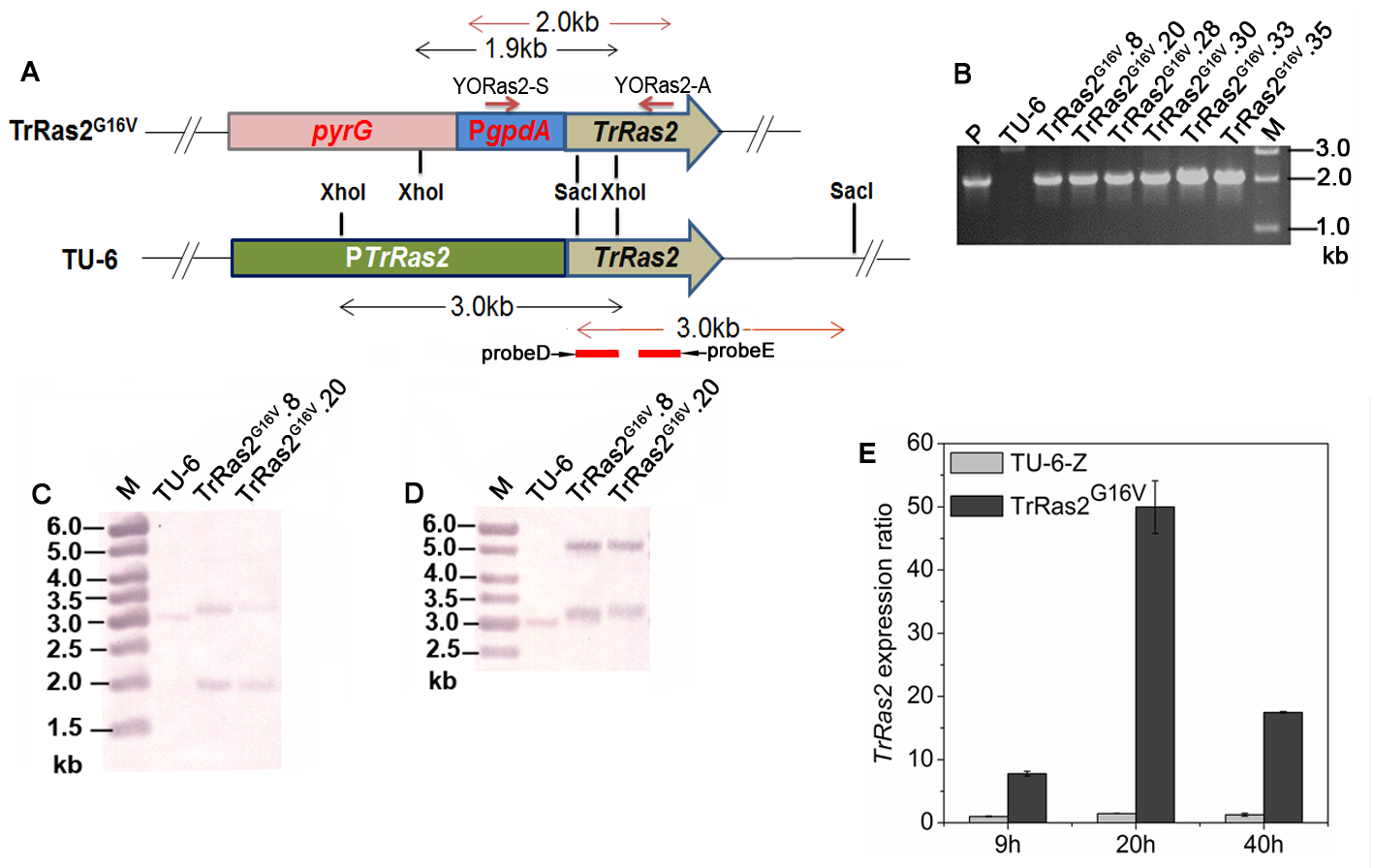

Supplement: Figure S4 — Construction of PAnigpdA - TrRas2G16V strains expressing the dominant activated TrRas2 allele. (A) Graphical representation of the TrRas2 genomic locus from the wild-type strain TU-6 and PAnigpdA-TrRas2G16V strains. Primer pairs and relative positions of the SacI and XhoI restriction sites are given. Probes D and E used for Southern analysis are shown as red boxes. (B) PCR analysis showed the successful integration of the pORas2 plasmid into the genome of TU-6. (C) Southern blot of the chromosome digested with XhoI confirmed the integration of the gpdA(p)-TrRas2G16V cassette into the genome of TU-6. (D) Southern blot of the chromosome digested with SacI confirmed that the gpdA(p)-TrRas2G16V cassette integrated ectopically. (E) Quantitative real-time PCR analysis showed that the TrRas2 mRNA levels in the PAnigpdA-TrRas2G16V strain was significantly increased when compared to that of the wild-type strain. The relative mRNA levels were presented by setting the amount of TrRas2 mRNA at 9 h in wild-type strain as 1. Actin gene was used as the reference. (TIF) [file pone.0048786.s004.tif]

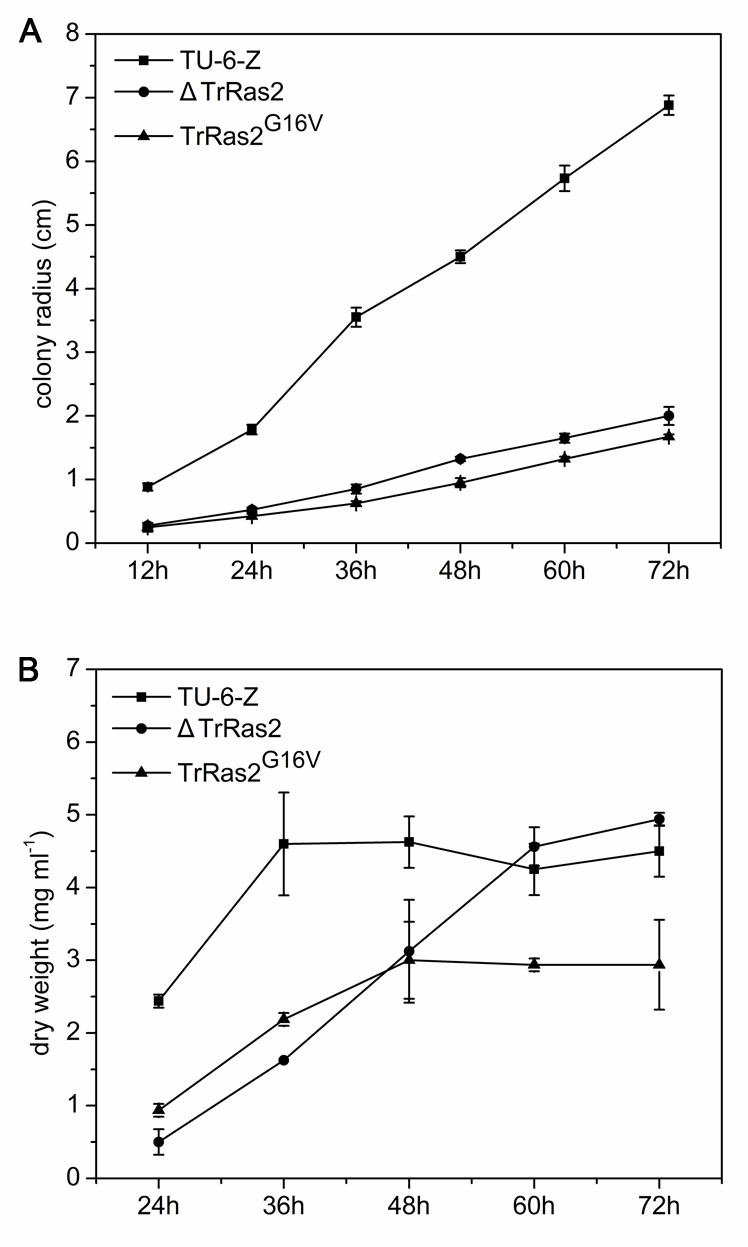

Supplement: Figure S5 — Radial growth and biomass of the TU-6-Z, Δ TrRas2 and PAnigpdA-TrRas2G16V strains. (A) Radial growth rates of the TrRas2 mutants. Equivalent squares of agar with growing strains were cultured on PDA plates at 30°C. (B) Biomass accumulation in LMM at 200 rpm and 30°C with equivalent squares of agar with growing relevant strains as the inoculation. (TIF) [file pone.0048786.s005.tif]

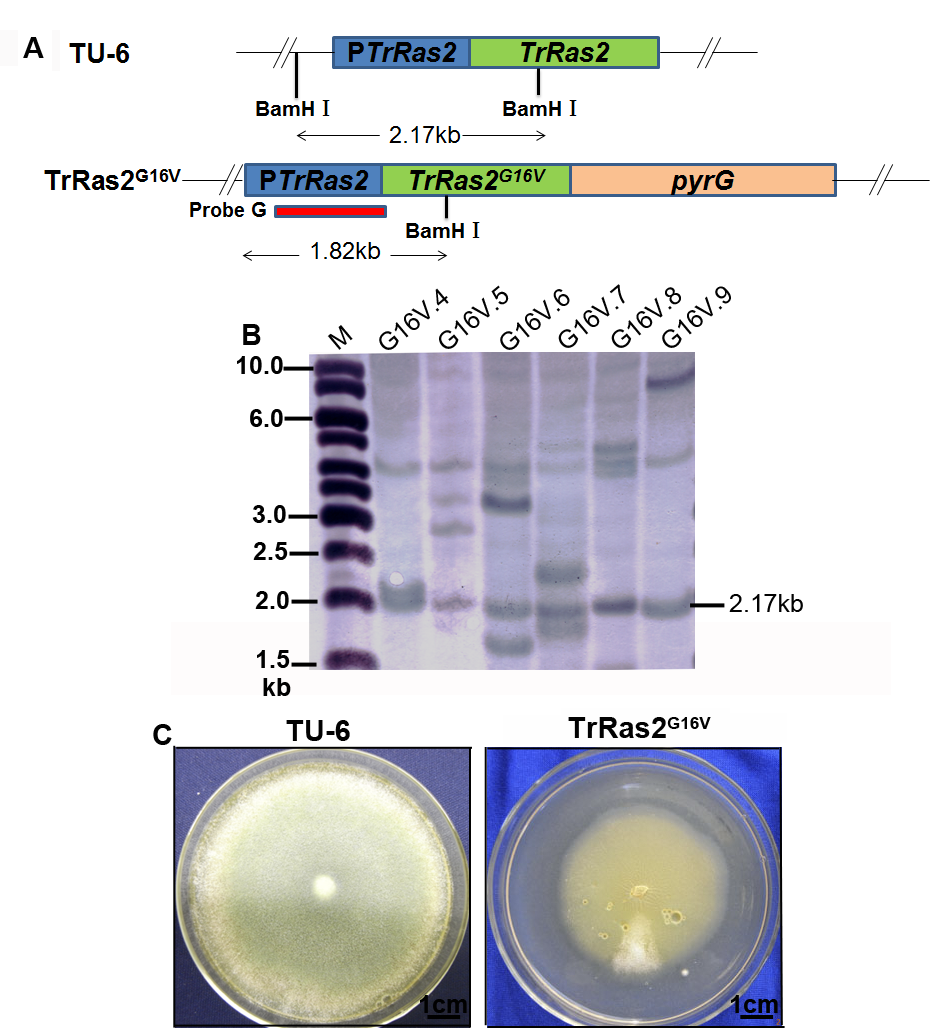

Supplement: Figure S6 — Construction of the dominant active TrRas2G16V mutant under the control of its own promoter. (A) Schematic representation of the genomic organization of the TrRas2 locus in the TU-6 and TrRas2G16V strains. Relative positions of the BamI restriction sites are given. Probe G used for Southern analysis is shown as red box. (B) Southern blot of the chromosome digested with BamI confirmed that the TrRas2G16V::pyrG+ cassette integrated ectopically. (C) Growth phenotypes of strains TU-6-Z and TrRas2G16V on PDA plates. One TrRas2G16V mutant was chosen as the representative. Scale bar = 1 cm. (TIF) [file pone.0048786.s006.tif]

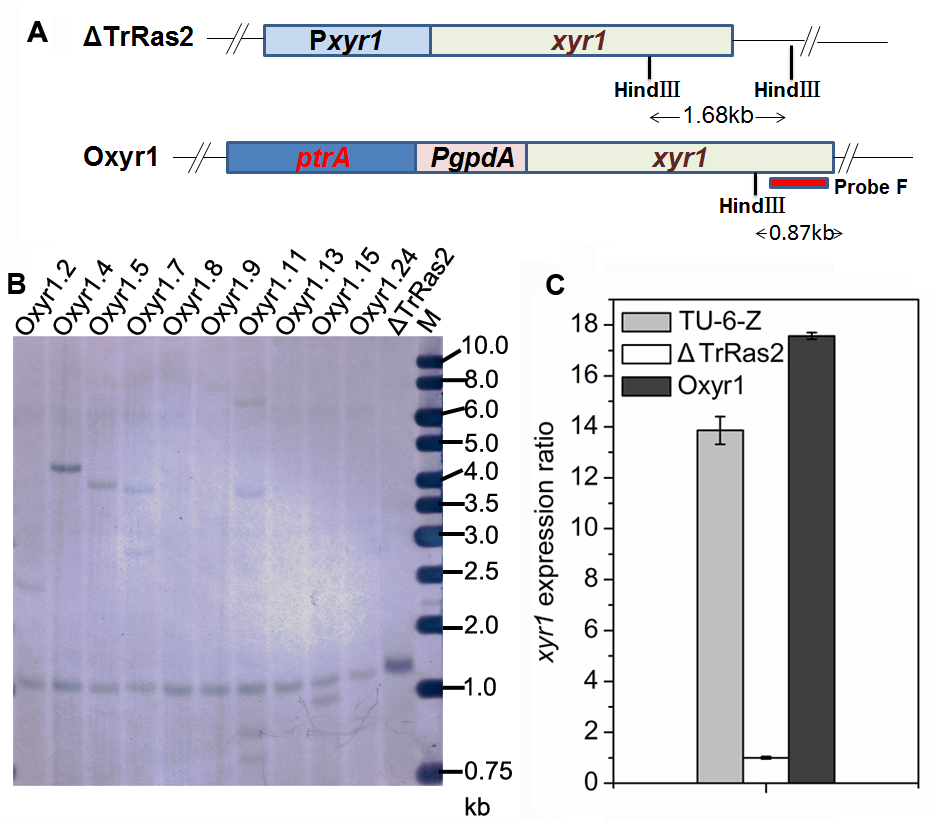

Supplement: Figure S7 — Overexpression of Xyr1 in the Δ TrRas2 mutant. (A) Schematic representation of the genomic organization of the xyr1 locus in the ΔTrRas2 and Oxyr1 strains. Relative positions of the HindIII restriction sites are given. Probe F used for Southern analysis is shown as red box. (B) Southern blot of the chromosome digested with HindIII. The 1.68 kb wild-type band is present in all the strains. An additional band longer than 0.87 kb indicates the presence of Oxyr1::ptrA + cassette. (C) Quantitative real-time PCR analysis of xyr1 in theTU-6-Z, ΔTrRas2 and Oxyr1 strains. Strains were induced on 1% Avicel cellulose for 22 h. The relative mRNA levels were presented by setting the amount of xyr1 mRNA in ΔTrRas2 as 1. Actin gene was used as the reference. (TIF) [file pone.0048786.s007.tif]
