# Supplementary material for: Ras GTPases Modulate Morphogenesis, Sporulation and Cellulase Gene Expression in the Cellulolytic Fungus Trichoderma reesei
Source: PLoS One. 2012 Nov 9;7(11):e48786. doi: 10.1371/journal.pone.0048786 (PMC3494722; doi:10.1371/journal.pone.0048786)
Supplement: Text S1 — Expression of the dominant active TrRas2G16V allele under control of its own promoter. (DOCX) [file pone.0048786.s008.docx]

Expression of the dominant active *TrRas2^G16V^* allele under control of its own promoter

For expression of a constitutively activated version of TrRas2^G16V^ under its own promoter, the cassette *TrRas2^G16V^*::*pyrG*^+^ containing 1.3 kb upstream of the predicted *TrRas2* translational start codon was constructed. The 5’ portion and 3’ portion of *TrRas2* were amplified using primer pairs Ras2-nest-S/O5Ras2-A and O3Ras2-S/ Dras2G16V-5-A respectively, which contains the mutation to be introduced into the wild-type template *TrRas2* DNA. A 2.7 kb *pyrG* fragment from pAB4-1 was generated using primer pair pyrG-S/pyrG-A. Then these three fragments were fused to generate the 5.9 kb *TrRas2^G16V^*::*pyrG*^+^ cassette using primer pair G16V-nest-S1/G16V-nest-A1. The *TrRas2^G16V^*::*pyrG*^+^ cassette was used for transformation of *T .reesei* TU-6. The integration and copy number of *TrRas2^G16V^* allele in the genome of *T. reesei* were analyzed by hybridization of *Bam*I-digested genomic DNA with PCR probe G generated using primer pair G16V-nest-S1/ O5Ras2-A. A 2.17 kb band was detected in the wild-type strain, and ectopic integration of the

*TrRas2^G16V^*::*pyrG*^+^ cassette lead to an additional hybridizing fragment longer than 1.82 kb (as shown in Figure 6A and B).

Morphological phenotypes of the *TrRas2^G16V^* mutants on PDA plates were analyzed, and the results showed that the *TrRas2^G16V^* stains displayed reduced colonies with no aerial hyphae and conidia, while their colonies exhibited regular borders, which is consistent with that in *PAnigpdA*-*TrRas2^G16V^* strains (Figure S6C). These results indicated that the phonotypical changes in the *PAnigpdA*-*TrRas2^G16V^* strains were attribute to the activated form of *TrRas2^G16V^* but not the overexpression of this gene.
