# Supplementary material for: Ras GTPases Modulate Morphogenesis, Sporulation and Cellulase Gene Expression in the Cellulolytic Fungus Trichoderma reesei
Source: PLoS One. 2012 Nov 9;7(11):e48786. doi: 10.1371/journal.pone.0048786 (PMC3494722; doi:10.1371/journal.pone.0048786)
Supplement: Table S1 — Primers used in this study. (DOCX) [file pone.0048786.s009.docx]

**Table S1 Primers used in this study. Restriction sites are underlined.**

| Primers | Sequences (5’-3’) | employment |
| --- | --- | --- |
| Ras1-5-S | AGAAGCGTTATCATCCGTAGTTG | mutant construction or probe |
| Ras1-5-A | GAAGCATAAAGTGTAAAGCCTGGGGACTAGGCTGGGACAAGACACG | mutant construction or probe |
| Ras1-3-S | AATACAAACAAAGATGCAAGAGCGGTGATGACTAGAGGCGACATTC | mutant construction |
| Ras1-3-A | CATAGGCGTTCTGGAGTATTGTA | mutant construction |
| ptrA-S | CCCCAGGCTTTACACTTTAT | mutant construction |
| ptrA-A | CCGCTCTTGCATCTTTGTT | mutant construction |
| Ras1-nest-S | TGAACGTCTACACCGCACTT | mutant construction |
| Ras1-nest-A | TAGGCGTTCTGGAGTATTGTA | mutant construction |
| YΔRas1-S | GCCCGGTACGAAGCACCTGTA | mutant construction |
| YΔRas1-A | CCAAGTCAGAACCCGACTCCC | mutant construction |
| Ras2-5-S | AATGCGTGCGAGAATTATAGACC | mutant construction |
| Ras2-5-A | CTTGCGGAGACGAGACACGACGGCGGCAGAAGCAGAAGCAAGAGGC | mutant construction or probe |
| Ras2-3-S | ACCAGATAACATTGACCAGCGGCTCACCCTCTGTGCTTCTCCTCAT | mutant construction |
| Ras2-3-A | AAATGGTCCAAAGTCAGCAAGA | mutant construction |
| pyrG-S | CGCCGTCGTGTCTCGTCT | mutant construction |
| pyrG-A | AGCCGCTGGTCAATGTTATC | mutant construction |
| Ras2-nest-S | TGTCAATGGATGGATGCCTGTAGTC | mutant construction or probe |
| Ras2-nest-A | TGGTGATAACGAGTAGAGAAGGGGA | mutant construction |
| YΔRas2-S | GCCCATTGCTCGCACCATCT | mutant construction |
| YΔRas2-A | TGCGCCCGTAGGTGGTTCGT | mutant construction |
| Rras2-S | TACAAACAAAGATGCAAGAGCGGTGTCAATGGATGGATGCCTGTAGTC | mutant construction |
| Rras2-nest-S | CGGCTCGTATGTTGTGTGGAAT | mutant construction |
| Rras2-nest-A | ACCAAAAGGGACTCCTGGAACAC | mutant construction |
| TPRas1-5-A | CAGCGGCACCCGATTCTATCCGTTCCTT-CGAGCTGCTGCTGTTTCTGTG | mutant construction |
| APcbh1-nest-S | AAGGAACGGATAGAATCGGGTGC | mutant construction |
| Pcbh1-S | AACAAAGATGCAAGAGCGGCAAAGCGTTCCGTCGCAGTA | mutant construction |
| Pcbh1-A | CAGTCCGCGGTTGACTATTGG | mutant construction |
| TPRas1-3-S | GAAACCCAATAGTCAACCGCGGACTGAGTCTCGTTCACAATGGCTACA | mutant construction |
| TPRas1-3-A | CAGATAGGAGATGCGAGTTTGTAG | mutant construction |
| TPRas1-nest-A | AAGCGTTTGTTGTCAGATGAGTTTG | mutant construction |
| YTPRas1-S | TTGACATGCACCTCAGACACG | mutant construction |
| YTPRas1-A | TTTACATTGTCGGTTGGTTTGG | mutant construction |
| O5Ras2-A | TTACCGACACCAACGTCTCCCAGCA | mutant construction or probe |
| O3Ras2-S | TGCTGGGAGACGTTGGTGTCGGTAA | mutant construction |
| PgpdA-S | GCGCGAATTCAGACCTAATACAGCCCCTAC (*Eco*RІ) | mutant construction |
| PgpdA-A | GCGCGGTACCTGTCTGCTCAAGCGGGGTAG | mutant construction |
| pyrG-*Eco*RI-S | GCGCGAATTCCGCCGTCGTGTCTCGTCT (*Eco*RІ) | mutant construction |
| pyrG-*Eco*RI-A | GCGCGAATTCAGCCGCTGGTCAATGTTATC (*Eco*RІ) | mutant construction |
| YORas2-S | GGCAGTAAGCGAAGGAGAATGT | mutant construction |
| YORas2-A | AATCGAGGAGCCGTGAGGTT | mutant construction |
| Ras1-probe-s | GCAAGCCACTCGAGCCGCTGAAG | probe |
| Ras2G16V-probe-S | GACGTTGGTGTCGGTAAGACG | probe |
| Ras2G16V-probe-A | CGACCTGCTTGCGGTATGAGT | probe |
| Ras2-inner-S | TACCGCAAGCAGGTCGTCATCG | probe |
| Ras2-inner-A | GCACTTGGACTTGCCCTTCTCG | probe |
| real-cbh1-S1 | GGTGGCGTGAGCAAGTATCC | real-time PCR |
| real-cbh1-A1 | TGTCCTCCAATGCCCGTGTT | real-time PCR |
| real-cbh2-S2 | CTGGTCCAACGCCTTCTTCA | real-time PCR |
| real-cbh2-A2 | GACCCAGACAAACGAATCCAG | real-time PCR |
| real-xyr1-S1 | TCTCCGTCGCTATTCTGCC | real-time PCR |
| real-xyr1-A1 | ATCATGCGTGAACTCGAAAGC | real-time PCR |
| real-ace1-S2 | GGACGAGGAGGAGATTATG | real-time PCR |
| real-ace1-A2 | GTGAGTCTTCTCGTGCTT | real-time PCR |
| real-ace2-S1 | CCCCAAGTTTGCGGTTCCT | real-time PCR |
| real-ace2-A1 | GCTCGTTCCAGTAAAATCCCA | real-time PCR |
| real-cre1-S2 | AGGCACGCCAAGAGGTCAA | real-time PCR |
| real-cre1-A2 | GGACAGGTTTCTCAGACTCGG | real-time PCR |
| real-ras1-S1 | CGGTGGTGGTGTCGGTAAA | real-time PCR |
| real-ras1-A1 | GGAGTAGACCAGCAAGAAACCC | real-time PCR |
| real-ras2-S1 | CTCGTCTACAGCATCGCATCC | real-time PCR |
| real-ras2-A1 | GGTGACCCTGTCGCTCTTGTT | real-time PCR |
| real-actin-S | CCCAAGTCCAACCGTGAGA | real-time PCR |
| real-actin-A | GAATGGCGTGAGGAAGAGC | real-time PCR |
| Ras1-RT-S | AGTCTCGTTCACAATGGCTACAGCA | RT-PCR |
| Ras1-RT-A | CTTGCACAGCAAAGGCTCTCACATG | RT-PCR |
| Ras2-RT-S | ATGGCGGGCAGAATGGTGCTGTACA | RT-PCR or mutant construction |
| Ras2-RT-A | GGAAGCGGCAACGAATGAGAA | RT-PCR |
| Oxyr1-001 | AGTATAATACAAACAAAGATGCAAGAGCGGAGACCTAATACAGCCCCTAC | mutant construction |
| Oxyr1-002 | AACAGCTACCCCGCTTGAGCAGACAATGTTGTCCAATCCTCTCCGTC | mutant construction |
| Oxyr1-003 | ATCACTGCCAAACGCACAAAC | mutant construction |
| Oxyr1-004 | CTACCCATTACCGTGGTTATCTG | mutant construction or probe |
| Oxyr1-008 | TTTAGCAAGGTTATGCGAAGC | probe |
| Dras2G16V-5-A | GCGGAGACGAGACACGACGGCGTGGTGATAACGAGTAGAGAAGGGGA | mutant construction |
| G16V-nest-S1 | CTCGCTGTCCAGTGCGTTTA | mutant construction or probe |
| G16V-nest-A1 | CCGCTGGTCAATGTTATCTGGT | mutant construction |
| NT7-CBH1-S | GTGCGGCTTGAACGGAGCTCTC | probe |
| NT7-CBH1-A | TAATACGACTCACTATAGGGATCATCCCACAGACTCATGACCAGA | probe |
